# SARS-CoV-2 peptide fragments selectively dysregulate specific immune cell populations via Gaussian curvature targeting

**DOI:** 10.1073/pnas.2521841122

**Published:** 2026-01-08

**Authors:** Yue Zhang, Carlos Silvestre-Roig, Han Fu, Haleh Alimohamadi, Taraknath Mandal, Jonathan W. Chen, Elizabeth Wei-Chia Luo, Jaime de Anda, Anna Lívia Linard Matos, Mathis Richter, Anna Mennella, HongKyu Lee, Liana C. Chan, Yingrui Wang, Naixin Wang, Hongyu Wang, Xiaohan Wang, Calvin K. Lee, Susmita Ghosh, Tsutomu Matsui, Thomas M. Weiss, Tiannan Guo, Maomao Zhang, Dapeng Li, Matthew C. Wolfgang, Robert S. Hagan, Melody M. H. Li, Matthias Gunzer, Albert Sickmann, Loredana Frasca, Michael R. Yeaman, Roberto Lande, Qiang Cui, Oliver Soehnlein, Gerard C. L. Wong

**Affiliations:** ^a^Department of Bioengineering, University of California, Los Angeles, CA 90095; ^b^Department of Chemistry and Biochemistry, University of California, Los Angeles, CA 90095; ^c^Department of Microbiology, Immunology and Molecular Genetics, University of California, Los Angeles, CA 90095; ^d^California NanoSystems Institute, University of California, Los Angeles, CA 90095; ^e^Material Science and Engineering, School of Engineering, Westlake University, Hangzhou, Zhejiang 310012, China; ^f^Research Center for Industries of the Future, Westlake University Hangzhou, Zhejiang 310024, China; ^g^Institute of Experimental Pathology, Center for Molecular Biology of Inflammation, University Hospital Münster, University of Münster, Münster 48143, Germany; ^h^Department of Chemistry, Boston University, Boston, MA 02215; ^i^Department of Physics Engineering, Boston University, Boston, MA 02215; ^j^Department of Biomedical Engineering, Boston University, Boston, MA 02215; ^k^Department of Physics, Indian Institute of Technology Kanpur, Kanpur 208016, India; ^l^Istituto Superiore di Sanità, National Center for Global Health, Roma 00161, Italy; ^m^Division of Molecular Medicine, Harbor-University of California Los Angeles Medical Center Los Angeles County, Torrance, CA 90502; ^n^Affiliated Hangzhou First People’s Hospital, Westlake University, Hangzhou, Zhejiang 02215, China; ^o^State Key Laboratory of Medical Proteomics, Hangzhou, Zhejiang 02215, China; ^p^School of Medicine, Westlake University, Hangzhou, Zhejiang 02215, China; ^q^Department of Cardiology, The Second Affiliated Hospital of Harbin Medical University, Harbin, Heilongjiang 150081, China; ^r^School of Life Sciences, Westlake University, Hangzhou, Zhejiang 310012, China; ^s^Center for Infectious Disease research, Westlake University, Hangzhou, Zhejiang 310024, China; ^t^Leibniz-Institut für Analytische Wissenschaften, Dortmund 44139, Germany; ^u^Stanford Synchrotron Radiation Lightsource, SLAC National Accelerator Laboratory, Stanford University, Menlo Park, CA 94025; ^v^Department of Microbiology and Immunology, University of North Carolina School of Medicine, Chapel Hill, NC 27599; ^w^Marsico Lung Institute, The University of North Carolina at Chapel Hill School of Medicine, University of North Carolina at Chapel Hill, Chapel Hill, NC 27599; ^x^Division of Pulmonary Diseases and Critical Care Medicine, The University of North Carolina at Chapel Hill School of Medicine, University of North Carolina at Chapel Hill, Chapel Hill, NC 27599; ^y^Institute for Experimental Immunology and Imaging, University Hospital, University of Duisburg-Essen, Essen 45141, Germany; ^z^Medizinisches Proteom-Center, Ruhr-Universität Bochum, Bochum 44801, Germany; ^aa^Division of Infectious Diseases, Harbor-University of California Los Angeles Medical Center Los Angeles County, Torrance, CA 90502; ^bb^Department of Medicine, David Geffen School of Medicine, University of California, Los Angeles CA 90095; ^cc^Institute for Infection and Immunity, Lundquist Institute for Biomedical Innovation, Harbor-University of California Los Angeles Medical Center, Torrance, CA 90502

**Keywords:** immune cell dysregulation, membrane permeation, antimicrobial peptides, membrane elasticity theory, COVID-19

## Abstract

Immune cell populations are dysregulated in COVID-19 for currently unknown reasons: Plasmacytoid dendritic cell (pDC) populations are reduced, thus hampering antiviral responses. CD8^+^ T cell populations are reduced, the level of which has emerged as an index of disease severity. Recent work has shown that the proteome of SARS-CoV-2 is a rich reservoir of antimicrobial peptide-like sequence motifs (xenoAMPs) which can chaperone and organize dsRNA for amplified Toll-Like Receptor 3 (TLR3)-mediated inflammation in vitro and in vivo. Here, we demonstrate that proteolytic digestion of the SARS-CoV-2 spike protein by host trypsin-like serine proteases directly produces xenoAMPs. Synchrotron Small Angle X-ray Scattering, mass spectrometry, and a theoretical analysis based on continuum membrane elasticity show that proteolytically generated xenoAMPs from SARS-CoV-2 proteins in vitro and machine learning-predicted high-scoring xenoAMPs all induce negative Gaussian curvature (NGC) necessary for pore formation in membranes. We find that xenoAMPs alone as well as xenoAMPs synergistically with endogenous AMP LL-37 can induce NGC in membranes. A computational analysis of immune cells with morphologically complex shapes (e.g., pDC, CD8^+^, and CD4^+^ T cells) suggests that surfaces with high local NGC can concentrate AMP-like sequences and promote selective membrane disruption. Consistent with this hypothesis, experiments with freshly isolated human peripheral blood mononuclear cells confirm that viable pDCs, DCs, and T cells are significantly depleted after xenoAMP exposure, in contrast to monocytes and neutrophils, the immune cell subsets with spheroidal morphology. Structural data from Omicron variant xenoAMP homologs indicate reduced pore formation, consistent with clinical observations of reduced T cell cytopenia in Omicron variant infections.

## Significance

Previous work has demonstrated that the proteome of SARS-CoV-2 can potentially be a rich source of AMP-like viral fragments, exemplars of which are associated with severe COVID-like inflammation in vitro and in vivo. Here, we demonstrate that direct proteolytic processing of SARS-CoV-2 proteins can yield xenoAMPs, and that the full heterogeneous ensemble of resultant fragments can collectively exert AMP-like pore-forming activity. We describe an unanticipated general mechanism of host cellular targeting for viral AMP-like pore forming peptides, based on local Gaussian curvatures of the host cell membrane, and show that this mechanism can selectively target and deplete specific immune cell types in a manner consistent with clinical observations for severe COVID-19 patients.

In most viral infections, appropriate molecular and cellular responses are orchestrated to achieve viral clearance. Plasmacytoid dendritic cells (pDCs) detect viral components and initiate innate responses including type I and type III interferons (IFNs) and cytokines to activate adjunctive immune cell types such as CD4^+^ and CD8^+^ T cells, as well as natural killer (NK) cells and B cells. Antigen-presenting cells displaying viral peptides activate T cells and prime them for killing of infected cells (e.g., CD8^+^ T cell cytolysis). In turn, monocyte/macrophage populations expand and become strongly activated to promote antiviral defense. In severe cases of COVID-19, this orchestration of protective immune responses is drastically different: Innate responses are dysregulated, and neutrophil mediated inflammation is profusive and protracted, resulting in off-target inflammation that damages bystander tissue. A profound alteration is also observed in specific host immune cell populations: Lymphocyte (including CD4^+^ T cell, CD8^+^ T cell, B cell) and NK cell counts are significantly reduced in peripheral blood in COVID-19 pneumonia for both mild and critical cases (1). In fact, CD8^+^ T cell depletion has emerged as the most robust indicator of COVID-19 disease severity (2). Similarly, rapid depletion is observed in pDC cell subsets, which normally act as “first responders” in viral infection through fast and potent early interferon signaling to activate downstream antiviral IFN-stimulated genes (ISGs). Such cellular depletion can persist for up to seven months in both hospitalized and nonhospitalized patients (3). In contrast, monocyte populations were not significantly different between COVID-19 patients and normal healthy individuals (4). Despite extensive research on COVID-19, an understanding of the mechanisms driving the selective deficiency of antiviral immune cells remains incomplete.

Recent work has shown that the proteome of SARS-CoV-2 but not those of common cold coronaviruses is a rich source of “xenoAMPs,” antimicrobial peptide (AMP)-like sequence motifs. Once released from the viral protein during proteolytic degradation, they can chaperone and organize dsRNA for amplified Toll-Like Receptor (TLR)-3 activation, resulting in elevated proinflammatory cytokine secretion in diverse cell types in culture (epithelial cells, endothelial cells, monocytes, and macrophages), a COVID-19 like transcriptome from endothelial cells, and a significant boost in plasma levels of IL-6 and CXCL1 in mice (5). While protease activity can in principle degrade viral proteins and thereby in principle suppress infection processes, certain protease families were found to promote infection. For example, the KLK family of trypsin-like serine proteases was found to facilitate influenza and betacoronavirus (SARS-CoV-2 and MERS) viral entry by cleaving and activating the viral fusion protein (6). A general picture of how proteolytic products of viral proteins interact with the immune system, however, has not been systematically investigated.

Here, we examine the possibility that a subpopulation of SARS-CoV-2 viral fragments generated from host proteolytic processing can selectively suppress specific immune cell populations via an unanticipated form of host cell targeting based on detection of membrane Gaussian curvature. The quantity and distributions of xenoAMP sequences in SARS-CoV-2 infected hosts are expected to be heterogeneous and correlated to specific enzyme proteome (enzome) of the infected host. What’s more, since many AMPs preferentially permeate bacterial membranes over mammalian membranes (7), higher local AMP concentrations are in general required to impact host cells. The studies here are designed to be a first exploration of these hypotheses. Using a combination of Liquid Chromatography with tandem mass spectrometry (LC-MS/MS) and machine learning, we find that viral fragments generated by tryptic digestion of SARS-CoV-2 spike proteins include AMP-like sequences (“xenoAMPs”). The propensity for xenoAMP production from viral proteins is enhanced by the enrichment of cationic residues in the SARS-CoV-2 proteome (5), since cationic charge is one of the defining characteristics of AMP sequences. This process of xenoAMP generation is cognate to the production of human AMPs, as exampled by the activation of human AMPs LL-37 by the protease kallikrein KLK5 (8).

We show using Small angle X-ray scattering (SAXS) and molecular dynamics (MD) simulations that xenoAMPs can mimic the ability of host AMPs to induce negative Gaussian curvature (NGC) in lipid membranes, which is a geometric requirement for forming transmembrane pores. This membrane remodeling ability is observed for xenoAMPs generated in a broad range of distinct contexts, including specific xenoAMPs generated from direct tryptic digestion of the SARS-CoV-2 spike protein, synthesized xenoAMPs that exhibit high predicted σ-scores in our validated machine learning classifier for identifying AMP-like sequences (9–11), and the peptide mixture released during trypsin or KLK5 digestion of spike protein. XenoAMPs can induce NGC *and* can enhance the pore-forming activity of endogenous AMP LL-37 via a “division of labor” mechanism. Consistent with these results, Radial Diffusion Assays (RDAs) indicate that these SARS-CoV-2 xenoAMPs have broad spectrum antimicrobial activity against *Acinetobacter baumannii*, *Escherichia faecalis*, *Klebsiella pneumoniae*, and the fungus *Candida albicans*, with antimicrobial activity levels quantitatively similar to typical AMPs. Using a mean field elastic theory, we estimate a xenoAMP(S) induced transmembrane pore size of 1.46 to 1.86 nm from SAXS measurements, a size which is comparable to that generated by the AMP-like N-terminal fragment of histone H4, previously shown to be capable of inducing lytic cell death of endothelial cells (11), in agreement with MD simulations. Based on computational estimates of xenoAMP spatial distributions on membrane regions with different local curvatures, we propose a model whereby xenoAMPs (and by implication AMPs) preferentially partition to membrane regions with strong local NGC, such as the septum of dividing bacterial cells (12), the microvilli-rich morphology of activated T cells or the “spiky” morphology of activated pDCs. Last, we directly examine the effect of these SARS-CoV-2 viral xenoAMPs on different immune cells that are freshly isolated human peripheral blood mononuclear cells (PBMCs). We find that these xenoAMP motifs can preferentially target and kill microvilli-rich immune cells, including pDCs, CD4^+^ T cells, and CD8^+^ T cells with surprisingly high efficacy (3.2 to 64.5 times higher cell death) but not spheroidal monocytes or neutrophils, in a manner that recapitulates extant observations in severe COVID-19 patients. In a more general compass, these results suggest a general mechanism through which host cell types with specific morphologies can have enhanced vulnerability to AMP-like motifs.

## Results

### Tryptic Digestion of SARS-CoV-2 Spike Protein Produces Heterogeneous Fragments That Contain AMP-Like Subpopulations Capable of Pore Formation.

**Fig. 1.**
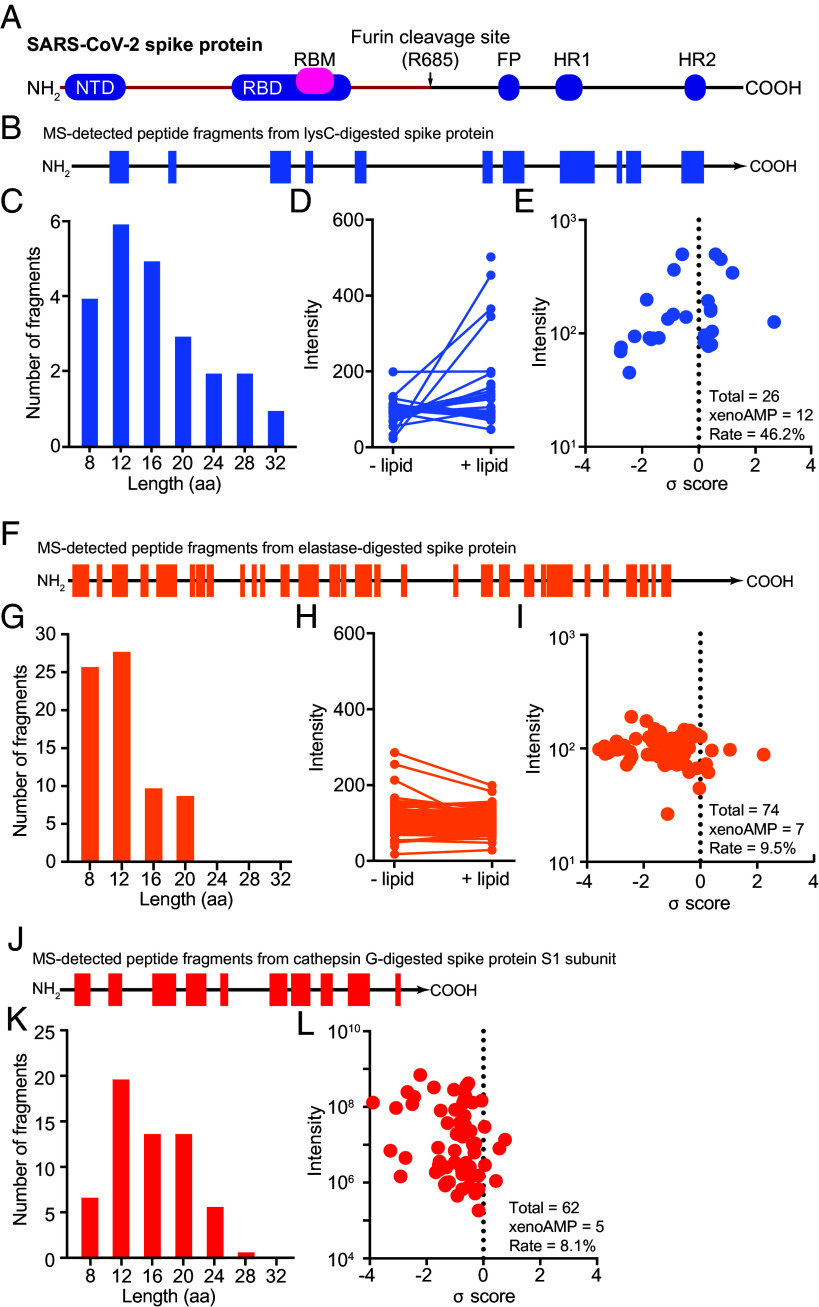
Trypsin-like serine protease cleaves SARS-CoV-2 spike protein into AMP-like peptides. (*A*) A diagram shows the function domains in SARS-CoV-2 spike protein: the N-terminal domain (NTD), The receptor binding domain (RBD), The receptor binding motifs (RBM), fusion peptide (FP), heptad repeat 1(HR1), and heptad repeat 2(HR2). The full length of the spike protein contains 1273 amino acids. The two subunits (S1 and S2) of spike protein are separated by a furin cleavage site (R685). (*B*) Full-length spike protein is digested with Lys-C. The peptide fragments are analyzed with LC-MS/MS. The location where the sequence is originally located in the protein is highlighted in blue. (*C*) The length distribution of peptides produced by Lys-C digestion. (*D*) Quantification of the peptides partitioned into the lipid phases. (*E*) Machine learning identification of xenoAMPs in the mixture of peptides produced by Lys-C digestion. A peptide with positive σ-score means the probability of being an AMP is above 0.5. (*F*–*I*) Analysis of the peptides produced by elastase-digested full-length spike protein. (*J*–*L*) Analysis of the peptides produced by cathepsin G-digested S1 subunit of spike protein.

SARS-CoV-2 is an enveloped single-stranded positive-sense RNA virus. The average number of SARS-CoV-2 virions in an infected host is estimated to be 10^9^–10^11^ (13). Due in part to their high copy numbers, SARS-CoV-2 proteins that are observed at the highest concentrations includes the spike protein, envelope protein, membrane protein, and the nucleocapsid proteins, the latter of which binds viral genomic RNA that eventually assembles into mature virions (14, 15). Indeed, viral proteins can persist in the host for months or even years in SARS-CoV-2 infections as well as for infections from other viruses (16, 17). These proteins are all, in principle, eventually proteolytically processed by the immune system (including proteolysis in service of epitope generation for antigen presentation). We explore these interactions by considering how SARS-CoV-2 spike protein is processed by three proteolytic activities known to be active in COVID-19. In order to assess the full spectrum of possible fragments from diverse serine proteases, we exploited both human and nonhuman commercially available serine proteases. These include Lys-C, elastase, and cathepsin G, which have different substrate specificities. (Fig. 1) Lys-C is model trypsin-like serine protease derived from *Lysobacter enzymogenes*. Lys-C’s strong and predictable lysine-specific proteolytic activity facilitates identification of the broad range of potential peptide fragments produced from the considerable repertoire of trypsin-like proteases. In contrast, elastase and cathepsin G are endogenous proteases stored in and released from host neutrophils, monocytes, and mast cells (18). Elastase cleavage targets C-terminal aspects of small, hydrophobic amino acids such as alanine, glycine, and valine. Cathepsin G cleavage targets C-terminal aspects of aromatic amino acids such as phenylalanine, tyrosine, and leucine. We examine how the SARS-CoV-2 spike protein (both the full-length version and the S1 subunit) are degraded into peptide fragments when they are incubated with the above proteases, using LC-MS/MS analysis.

All three proteases proteolytically digest SARS-CoV-2 spike proteins into short peptide fragments, but each protease produces a different distribution of peptide fragments, and with different sequence coverage (*SI Appendix*, Table S1). Digestion by trypsin-like serine protease Lys-C releases the least number of peptides, encompassing only 25.8% of the original protein sequence (Fig. 1*B*). In contrast, digestion by elastase and cathepsin G yield more peptide fragments with higher coverage of the native protein sequence (42.2% and 43.8%, respectively, Fig. 1*F* and *J*). The length distributions of the released peptides by three proteases are similar (Fig. 1*C*, *G*, and *K*). Importantly, Lys-C-generated peptide fragments have stronger affinity to the phosphatidylethanolamine (PE)-rich lipid membrane (Fig. 1*D*), a behavior often seen in AMPs (7). In contrast, peptides released by elastase digestion do not strongly partition into the PE-rich lipid. (Fig. 1*H*).

To determine whether any of the peptide fragments thus released during protease digestion exhibit physico-chemical properties similar to AMPs, we scan all LC-MS/MS-identified peptides with a machine learning-based AMP classifier. This classifier and its variations have correctly predicted various AMP-like peptides including neuropeptides PACAP (10), the N-terminal histone H4 fragment (11), and *Clostridioides difficile* enterotoxin fragments exhibit structure–function profiles consistent with AMP-like sequences (19). Each queried sequence is assigned a σ score to indicate it is “AMP-ness” Results indicate that all three proteases are capable of generating xenoAMPs when digesting spike proteins: The data in Fig. 1*E* is based on peptides with positive σ scores. For more stringent thresholds, we find that 8/26 fragments (30.8 %) from trypsin-like serine protease Lys-C are xenoAMPs with a σ score > 0.4 (σ score > 0.4 indicates a probability >79.4% of the sequence being able to function as an AMP). For elastase and cathepsin G, the fractions are lower, at 3/74 (4%) and 3/62 (4.8%) respectively (Fig. 1 *E*, *I*, and *L* and *SI Appendix*, Table S1). The subpopulation of xenoAMPs from digestion is substantial. Because xenoAMP predictions from the machine learning classifier are probabilistic in nature, it is necessary to determine whether heterogeneous AMP-like SARS-CoV-2 viral fragments produced by protease digestion experiments above can remodel membranes in a manner cognate to AMPs, even though they may not exhibit σ-scores as high as those from the predicted highest-scoring xenoAMPs.

### Specific xenoAMPs Proteolytically Generated From SARS-CoV-2 Spike Protein Can Induce Transmembrane Pores.

**Fig. 2.**
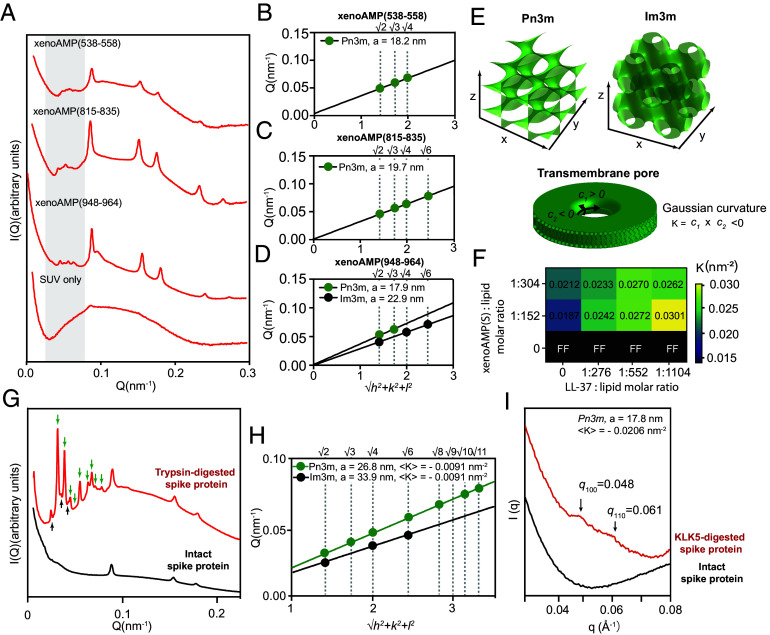
Individual Lys-C-generated xenoAMP, and the complete heterogeneous ensemble of peptides released from trypsin or KLK5-digested spike protein are all able to remodel membrane into NGC-rich cubic phases. (*A*) SAXS spectra from membrane incubated with three different xenoAMPs identified in Lys-C-digested spike protein respectively: xenoAMP(538-558) (σ = 0.6), xenoAMP (815-835) (σ = 1.19) and xenoAMP (948 to 964) (σ = 0.34). The membrane lipid composition is DOPS/DOPE/Cholesterol = 20/70/10. The peptide (or protein) to lipid molar ratio is P/L = 1/153. (*B*–*D*) Linear regression analysis shows the correlation peaks identified at the low q region are indexed into two bicontinuous cubic phases: Pn3m and Im3m. (*E*) Schematic representations of Pn3m and Im3m structures. Such cubic phases exhibit high concentrations of negative Gaussian curvature (NGC) per unit cell volume. NGC is topologically required for forming the transmembrane pores. (*F*) SUVs (DOPS/DOPE = 20/80) were incubated with xenoAMP(S) alone, LL-37 alone or with a mixture of xenoAMP(S) and LL-37 at varying P/L ratios. The Gaussian curvature was quantified SAXS-based characterization of the lipid crystal structure. (*G*) SAXS spectra of the SUV (DOPS/DOPE/Cholesterol=20/70/10) incubated with trypsin-digested spike protein or intact spike protein. The P/L molar ratio is 1/8000. (*H*) The characteristic correlation peaks found in trypsin-digested spike protein sample can be indexed into two cubic phases: Pn3m with lattice constant a = 26.844 nm (K = −0.0091 nm^−2^) and Im3m cubic phase with a lattice constant a = 33.878 nm (K = −0.0093 nm^−2^). (*I*) SAXS spectra of the SUV (DOPS/DOPE/Cholesterol=20/70/10) incubated with KLK5-digested spike protein or intact spike protein. P/L = 1/1000.

To investigate whether specific peptides released by tryptic proteolytic activity can induce transmembrane pore formation, an established bactericidal mechanism of AMPs, we select three representative xenoAMPs detected in Lys-C-digested spike protein samples: xenoAMP(538-558) (σ = 0.60), xenoAMP(815-835) (σ = 1.19), and xenoAMP(948-964) (σ = 0.34). Each xenoAMP is incubated with small unilamellar vesicles (SUVs) composed of a ternary mixture of phosphatidylserine (PS), PE, and cholesterol (Chl) in molar ratios of 20:70:10. The structure of the xenoAMP-induced lipid phases was analyzed using SAXS. Two distinct types of lipid phases were observed for all samples: 1) an inverse hexagonal phase (H_II_) with a lattice constant of ~8 nm (q-ratios √1: √3: √4: √7: √9), and 2) and bicontinuous cubic phases (Q_II_) (Fig. 2*A*).

In xenoAMP (538-558) samples, the indexed peaks show q ratios of √2: √3: √4, corresponding to a Pn3m cubic phase with a lattice constant of 18.2 nm (K = −0.02 nm^-2^) (Fig. 2*B*). For xenoAMP (815-835), the correlation peaks with q ratios of √2: √3: √4: √6 indicate a Pn3m cubic phase with a lattice constant of 19.7 nm (K = −0.017nm^−2^) (Fig. 2*C*). In the xenoAMP (948-964) sample, two sets of correlation peaks are observed at low q values. The first set (q ratios: √2: √4: √6) corresponded to an Im3m cubic phase with a lattice constant of 22.9 nm (K = −0.02 nm^−2^), while the second set (q ratios: √2: √3) indicated a Pn3m cubic phase with a lattice constant of 17.9 nm (K = −0.02 nm^−2^) (Fig. 2*D*).

Pn3m and Im3m are bicontinuous cubic phases rich in NGC, which is the class of curvature necessary for forming transmembrane pores (Fig. 2*E*). From the measured quantitative amount of NGC, we estimate transmembrane pore sizes to be in the range of 1.58 ± 0.20 nm, 1.48 ± 0.22 nm, and 1.60 ± 0.20 nm for xenoAMP(538 to 558), xenoAMP(815 to 835), and xenoAMP(948 to 964) respectively. The observation that the three xenoAMP sequences are all capable of inducing NGC suggests potential membrane permeation ability. Consistent with machine learning predictions and SAXS structural studies, antimicrobial experiments using radial diffusion assays (RDAs) show that these xenoAMPs exhibit unambiguous antimicrobial activity on bacteria and fungi, in a manner quantitatively similar to AMPs (*SI Appendix*, Table S2).

### Effects of Peptide Heterogeneity on Membrane Remodeling Activity.

Due to the nature of proteolysis and subsequent diffusive transport, we expect diverse membrane-active viral fragments (xenoAMPs) to coexist with one another, but also with non-membrane-active viral fragments (non-xenoAMPs). Moreover, given that an infected host can have varying degrees of inflammation, we also expect in some hosts the co-existence of “professional” host AMPs, such as LL-37. Each of these cases is examined in turn below. We find that heterogeneous populations of viral xenoAMPs and viral non-xenoAMPs induce NGC in membranes. Moreover, we find that heterogeneous populations of viral xenoAMPs and host LL-37 can play complementary roles and cooperatively induce NGC.

First, we examine what happens when two different membrane-active viral xenoAMPs act on a membrane. Different viral xenoAMPs can induce different curvatures in principle, so an assessment of collective effects is useful. As shown in *SI Appendix*, Fig. S1 *A* a mixture of two high-scoring xenoAMPs-xenoAMP(S) and xenoAMP(445 to 462) (identified in the spike protein via machine learning classifier)—collectively induce membrane curvature in a manner that generates larger pores than what either can do alone (20). These results are strongly suggestive: It is not necessary to have high concentrations of a specific single viral sequence to generate pores; a cohort of heterogeneous xenoAMP sequences with membrane activity can exert a population pressure to form pores in a manner cognate to AMPs.

Next, we examine whether viral xenoAMPs can synergize with host AMPs, given that different hosts can have different spatiotemporal availability of AMPs from preexisting inflammatory conditions. Human cathelicidin LL-37 is used here as a canonical example of an endogenous AMP. We find that viral xenoAMPs and LL-37 can cooperatively induce pore formation via an unexpected form of “division of labor”: In principle, there are many ways to promote pore formation by a specific peptide sequence. These include but are not limited to inducing specific types of curvature geometrically necessary for pores, decreasing the line tension of the pore, formation of topological defects in the membrane, induced thinning of the membrane. Fig. 2*F* shows the quantitative amount of negative Gaussian induced for different combinations of LL-37 and xenoAMP(S) at different relative concentrations. To highlight different roles played by LL-37 and xenoAMP(S), we use thermodynamic conditions where the LL-37 capacity for pore formation alone is not strong enough to induce three-dimensional (3-D) cubic phases (neutral pH, DOPS/DOPE = 20/80). Here, with no added xenoAMP(S), increases in the molar concentration of LL-37 relative to that of lipids induced no observable NGC-rich cubic phases, and only a typical lipid bilayer form factor is observed. In contrast, under the same conditions but with no LL-37, increases in the molar concentration of xenoAMP(S) leads to the unambiguous induction of NGC and cubic phases. In fact, with a baseline concentration of xenoAMP(S) added (P/L = 1/152 or 1/304), increases in the molar concentration of LL-37 leads to drastic increases in the NGC generated, in fact stronger increases than achievable from increasing xenoAMP(S) concentrations by itself. This cooperative effect suggests that xenoAMPs and inflammatory host pore-former like LL-37 (which may be derived from NETosis) may play different complementary roles in membrane disrupting activity. This can be particularly important in the respiratory tract of COVID-19 patients, where both LL-37 from neutrophil activation as well as SARS-CoV-2 proteins are abundant.

Finally, we contrast the NGC generating ability of the intact spike protein itself with that of peptide fragments generated from proteolytic digestion of the spike protein. Viral fragments released during proteolytic degradation comprise a diverse range of sequences, some of which exhibit physicochemical properties closely resembling AMPs (high-scoring xenoAMPs), while others do not. Here, we investigate what happens when cell membranes are exposed to the entire diverse population of viral fragments generated from proteolysis, which is composed of xenoAMPs and non-xenoAMPs, to see whether membrane remodeling activity is preserved. To answer this question, the spike protein is first digested into a mixture of peptide fragments by trypsin, which cleaves after both arginine and lysine. The fragmented spike protein and the intact spike protein are incubated with SUVs in separate experiments, and the remodeled membrane structure is analyzed using SAXS. Consistent with the SAXS results obtained with three exemplar xenoAMPs, three lipid phases coexist: 1) an inverse hexagonal phase (H_II_) (peaks with q-ratios of √1: √3: √4); 2) a Pn3m inverse bicontinuous cubic phase (Q_II_) with a lattice constant of 26.844 nm (K = −0.0091 nm^−2^). The q ratio of the correlation peaks is √2: √3: √4: √6: √8: √9: √10: √11; 3) a Im3m inverse bicontinuous cubic phase (Q_II_) with a lattice constant of 33.878 nm (K = −0.0091 nm^−2^). The positions of the correlation peaks are observed at q-ratios of √2: √4: √6 (Fig. 2*G*–*H*), although the protein to lipid molar ratio (1/8,000) used in this experiment is ~52 times lower than the ratio used in single xenoAMP experiment. Similar results are observed in a parallel experiment using KLK5, an epithelial-derived serine protease with trypsin-like substrate specificity. KLK5 was found to enhance influenza and human betacoronavirus (including SARS-CoV-2) infection via cleave and activate their surface glycoprotein (6, 21). SAXS analysis revealed that the ensemble of spike protein fragments released by KLK5 digestion remodel lipid membrane into a Pn3m cubic phase, as evidenced by characteristic scattering peaks at q-ratios of √2: √3 (Fig. 2*I*). The lattice constant is 17.8 nm and the NGC is −0.0206 nm^−2^. Taken together, these results confirm that the ability to induce NGC is preserved even in diverse peptide populations generated during proteolytic digestion, which comprise both xenoAMPs and non-xenoAMPs.

In an infected host, we expect significant heterogeneity in protease efficacy among different infected hosts, and in the spatiotemporal variation of protease concentrations in a given host (22, 23), which lead to more diverse and complex distributions of viral peptide fragments. In the above sections, we demonstrated that even in the limit of a single serine protease, xenoAMPs can be generated, and that heterogeneous peptide mixture can retain the ability to permeate membranes in a manner cognate to AMPs, an ability that can lead to host cell damage (11).

### A Direct Comparison Between Membrane Remodeling Capacity of SARS-CoV-2 xenoAMP(S) and of Histone H4 N-Terminal Fragment.

**Fig. 3.**
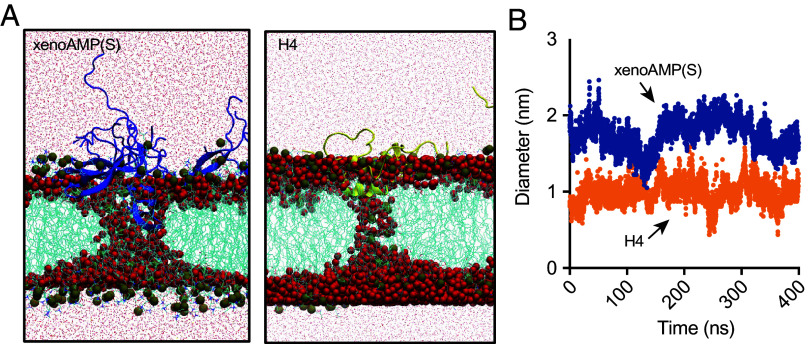
A comparison of membrane remodeling capacity between SARS-CoV-2 xenoAMP(S) and histone H4 N-terminal fragment. (*A*) An equilibrated structure of the transmembrane pore formed by xenoAMP(S) or histone H4 N-terminal fragment sampled in the MD simulation. xenoAMP(S) (blue) and histone H4 fragment (gold) are shown by cartoon representation. Lipids (cyan) are shown by line representation, and the phosphorus atoms are highlighted by gray spheres. Water inside and outside the membrane are highlighted by red spheres and dots, respectively. (*B*) Pore diameter near the pore center as a function of simulation time after formation of a stable water channel. xenoAMP(S) forms larger pore than histone H4 fragment. The latter one is a confirmed pore former on human endothelial cells.

The histone H4 N-terminal fragment has been shown to be able to induce lytic cell death in mammalian cells (more specifically, inducing smooth muscle cell death in atherosclerosis (11)). Indeed, histones are known to harbor a rich repertoire of AMP-like motifs, due to their high concentrations of Lys and Arg (24). To do a direct pore-forming activity comparison between the histone H4 fragment and xenoAMP(S), a sequence selected from the strongest AMP-like hotspot from the SARS-CoV-2 spike protein, we performed Molecular Dynamics (MD) simulations to visualize the interaction between xenoAMP(S) and the lipid bilayer composed of DOPS and DOPE at a molar ratio of 20:80. The results show multiple xenoAMP(S) strongly adsorb onto the lipid bilayer and stabilize the disordered toroidal membrane pore through a series of hydrophilic residues (e.g., THR547, THR549, and main chain groups in GLY548, GLY550) and the hydrophobic LEU546 residue on one of the β-strands (Fig. 3*A*), a phenomenon consistent with the NGC-stabilizing activity of xenoAMP(S) identified by SAXS (Fig. 2*F* and Fig. 5*A*). One of the xenoAMP(S) peptide inserts deeply inside the lipid bilayer and aligns with the water channel to stabilize the disordered toroidal membrane pore, and the rest of xenoAMP(S) lay close to edge of the pore, which is consistent with the structural features of disordered toroidal membrane pore formed by AMPs (25). Near the center of the transmembrane core, the flexible Asn-Gly kink is deeply inserted, indicating xenoAMP(S) stabilize the pore by adapting peptide shape to the water channel fluctuations. The diameter of the pore near the center is around 1.9 ± 0.2 nm, which is comparable with the pore formed by canonical AMP magainin. The pore size induced by xenoAMP(S) is comparable to the one induced by histone H4 fragments (Fig. 3*B*). Removing the xenoAMP(S) leads to the fast closure of the pore in 7ns, suggesting a strong pore-stabilization effect of xenoAMP(S). In *SI Appendix*, Fig. S1*B*, we show that other high-scoring xenoAMP fragments from other SARS-CoV-2 proteins can stabilize NGC and induce transmembrane pore formation.

### XenoAMPs Target Cell Membranes With High Local NGC.

AMPs’ membrane permeation activity is strongly influenced by the chemical and physical properties of the lipid membrane. Anionic lipid and lipid with intrinsic negative curvature promote the AMP-mediated transmembrane pore formation (7, 26). In Fig. 2 we show, like natural AMP, SARS-CoV-2 derived xenoAMPs can stabilize NGC on the model lipid membrane. We seek to study how the preexisting local membrane curvature affects the lateral phase separation of xenoAMPS on the surface. The effective concentration of xenoAMPs is important for understanding the strength of interactions between xenoAMPs and host immune cells. Here, we construct a simple model to study how local membrane geometry affects the accumulation of xenoAMPs on membrane. The membrane is modeled as an elastic thin sheet with xenoAMPs that freely diffuse on it (27). Assuming the system is in mechanical equilibrium and for low peptide concentration (weak peptide–peptide interactions), the free energy density of the system (W), including the mismatch elastic energy of membrane–peptide interactions and the entropic contributions from peptide diffusion on the membrane surface, is given by (28–30) (the complete derivations are provided in the *SI Appendix*)

[1] $$\begin{array}{c} W=\underset{\text{Membrane}-\mathrm{peptides}\;\text{interaction}\;\mathrm{energy}}{\underbrace{\left[\kappa H^{2}+\kappa {\left(D-D_{0}\phi \right)}^{2}\right]}}+\underset{\text{Entropy}\;\text{of}\;\text{peptide}\;\text{distribution}}{\underbrace{k_{B}Tn_{s}\left(\phi \;\log\left(\phi \right)+\left(1-\phi \right)\log\left(1-\phi \right)\right)}}, \end{array}$$

where κ is the membrane bending rigidity, $H=(C_{1}+C_{2})/2$ is the membrane mean curvature ($C_{1}$ and $C_{2}$ are the principal curvatures of the membrane at any given point), $D=(C_{1}-C_{2})/2$ is the membrane curvature deviator, and $D_{0}=\left(C_{1p}-C_{2p}\right)/2$ is the induced deviatoric curvature by xenoAMPs. $k_{B}$ is the Boltzmann constant, T is the absolute temperature, $n_{s}$ is the saturation density of xenoAMPs on surface ($n_{s}∼$ 1/area of peptide), and ϕ is the fraction of the membrane area covered by xenoAMPs. In Eq. **1**, we assume that at low peptide concentration, the contribution of peptide–peptide interaction energy is negligible compared to the elastic membrane–peptides interactions energy (31–33). We further assume that xenoAMPs primarily induce deviatoric curvature and the magnitude of the induced anisotropic curvature by xenoAMPs is linearly proportional to the peptide area fraction ϕ, which we estimate using our SAXS measurements (20, 34–36).

**Fig. 4.**
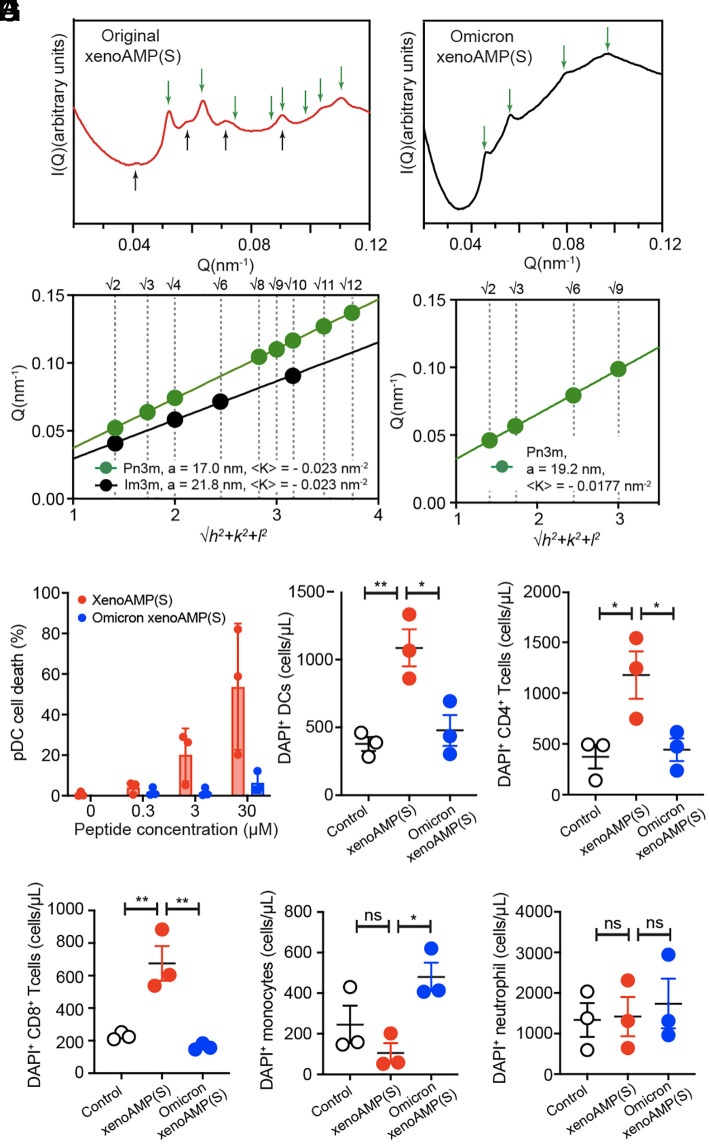
xenoAMPs preferentially accumulate on cell membrane regions enriched with NGC. (*A*) Heat map of xenoAMPs density (ϕ) as a function of membrane principal curvatures ($C_{1}$ and $C_{2}$). The model xenoAMP induces cubic phase lattice constant *a* = 10 nm. The modeling shows xenoAMP preferentially accumulates in domains with opposite principal curvatures (NGC, $C_{1}C_{2}<0$). (*B*) ϕ as a function of deviatoric curvature for a fixed $C_{1}$ = −1 nm^−1^. Three model xenoAMPs with different cubic lattice constants are studied. The magnitude of ϕ increases with increasing membrane deviatoric curvature, while a smaller lattice constant decreases the slope of the transition to ϕ=1. (*C*) A scheme depicting the surface morphology of monocytes and T cells (37). (*D*) Distribution of xenoAMPs in an idealized geometry of an activated T cell with tubular protrusion with a radius of r. The protrusion is connected to the spherical body via a rotationally symmetric, half catenoid-shape neck. xenoAMPs are strongly localized in the half catenoid and the tubular regions e.g., for fixed a=10 nm and r = 10 nm, $\phi_{\mathrm{catenoid}}/\phi_{\mathrm{sphere}}∼4$. (*E*) Density of xenoAMPs in the half catenoid, spherical body, tubular protrusion, and the spherical cap regions as a function of the radius of the protrusion (r). The density of xenoAMPs in the half catenoid and the tubular regions decreases with increasing the radius of the protrusion and is higher than in the spherical domains. (*F*) Schematic of an idealized geometry of a dividing bacterial cell with a catenoid-shaped neck of radius r_neck_. The inset shows the distribution of the fluorescence channel (Rhodamine-LL37) on a single dividing *E. coli* cell adapted from (12). (*G*) Spatial distribution of LL37 as an NGC-generating AMP, on the dividing bacterial surface for three different neck sizes. LL37 preferentially accumulates in the neck domain, and its concentration increases as the neck becomes narrower.

Based on this simple model, we find that the xenoAMPs’ local areal density is sensitive to local membrane geometry. Local domains with opposing principal curvatures or NGC ($C_{1}C_{2}<0$) attract more xenoAMPs as this geometry aligns with peptides’ preferred intrinsic curvature, thereby reducing the peptide–membrane interaction energy as described in Eq. **1** (Fig. 4*A*). As the magnitude of membrane deviatoric curvature increases, the surface density of xenoAMPs increases linearly and then saturates (Fig. 4*B*). The saturation rate depends on xenoAMP’s capability of inducing deviatoric curvature. The peptides that strongly induce deviatoric curvature accumulate more on membranes with high deviatoric curvature and less on membranes with low deviatoric curvature. In contrast, weak deviatoric curvature-inducing peptides show a more even distribution on membranes, independent of the amplitude of deviatoric curvature. From a physical perspective, the local accumulation of strong deviatoric curvature-inducing xenoAMPs can enhance their ability to permeate membranes more effectively.

We estimate the spatial distribution of xenoAMPs on the membrane of different immune cell types. For example, monocytes, neutrophils, and macrophages have simple convex shapes without large membrane protrusion. In contrast, activated dendritic cells and CD8^+^ T cells contain a large number of membrane protrusions in the form of spikes and microvilli respectively, which generate a gradient of curvature along these protrusions (37, 38) (Fig. 4*C*). For a microvillus, the tip of the microvillus is a cap dominated by positive Gaussian curvature; the middle section is a tube with zero Gaussian curvature, which connects to the cell body via a neck-shaped structure rich in NGC. For ease of modeling, we consider the idealized geometry of pDCs and T cells as a spherical body connected via a half catenoid-shaped transitional neck to a tubular protrusion (Fig. 4*D* and *SI Appendix*, Fig. S2*A*). Having a fixed number of xenoAMPs attached to the membrane ($\phi_{\mathrm{average}}=0.1$), our results show that xenoAMPs preferentially accumulate on the necks and the tubular protrusions with positive deviatoric curvature than in the spherical domains with zero deviatoric curvature. For example, for a narrow neck of r = 10 nm, the concentration of xenoAMPs in the neck domain is almost four times larger than the cell body (Fig. 4*E*). It is interesting that this distribution resembles the distribution of LL37 on a dividing *E. coli* cell, which showed that LL37 accumulates at the division neck region (Fig. 4*F* and *SI Appendix*, Fig. S2*B*) (12). Our results agree qualitatively with this trend: For an idealized geometry of a dividing bacterial cell, the NGC generating LL37 molecules preferentially localize at the division neck and its concentration significantly increases with increasing neck constriction (Fig. 4*G*). It should be noted that we have neglected the effect of orientational ordering and the resulting localization of topological defects, assuming that the associated segregation energy is negligible compared with elastic curvature interactions (39–42). However, the accumulation of negatively charged defects in regions of high NGC (such as the catenoid-shaped neck) can locally disrupt lipid ordering, lower the line tension of curvature-generated saddle pores, and thereby stabilize and facilitate membrane-pore formation by xenoAMPs. Together, these results suggest that the morphology of host cells in general and immune cells in particular play important roles in determining their vulnerability to xenoAMPs. We show that immune cells with regions rich in NGC concentrate more xenoAMPs and thereby are potentially more vulnerable to xenoAMP’s pore-forming activity.

### Omicron xenoAMPs Have Diminished Capacity for Inducing NGC On Lipid Membranes.

The SARS-CoV-2 Omicron variant has received significant attention since it emerged in November 2021 due to its high transmissibility and due to the alarmingly large number of mutations in the receptor binding domain (RBD). However, for reasons not entirely understood, the disease outcomes tend to be less severe compared to earlier strains of SARS-CoV-2. The Omicron variant’s spike protein harbors 37 mutations (43), primarily concentrated in the N-terminal domain (NTD) and receptor-binding domain (RBD), which enhances binding affinity to the human Angiotensin-converting enzyme 2 (ACE-2) and improve immune evasion by avoiding recognition from preexisting antibodies (e.g., N440K, G446S, etc). In contrast, only 8 mutations are found in the C-terminal domain (CTD) (T547K, H655Y, N764K, D796Y, N856K, Q954H, N969K, and L981F). The majority of these C-terminal mutations increase the overall positive charge. Some reports suggest that Omicron mutations reduce the spike protein’s fusogenicity and thus hinder viral entry (44), while other experimental results indicate that Omicron has increased replicative capacity and infectivity in nasal and airway organoids compared to the original SARS-CoV-2 strain (45), with patient nasal swabs showing comparable viral RNA levels across different variants (46). Thus, our understanding of how mutations in Omicron lead to reduced disease severity compared to the original strain remains incomplete.

Our results presented above suggest a parallel line of inquiry where we examine how mutations, especially mutations that increase cationic charge, in Omicron influence xenoAMP membrane remodeling behavior. Specifically, we compare the membrane permeation of xenoAMP(S) (the high scoring xenoAMP located at 529 to 558) with its Omicron homologous sequence carrying a single mutation T547K. The peptides are incubated with SUVs containing a binary mixture of DOPS and DOPE at a molar ratio of 20:80, and the resultant structure is analyzed using SAXS.

**Fig. 5.**
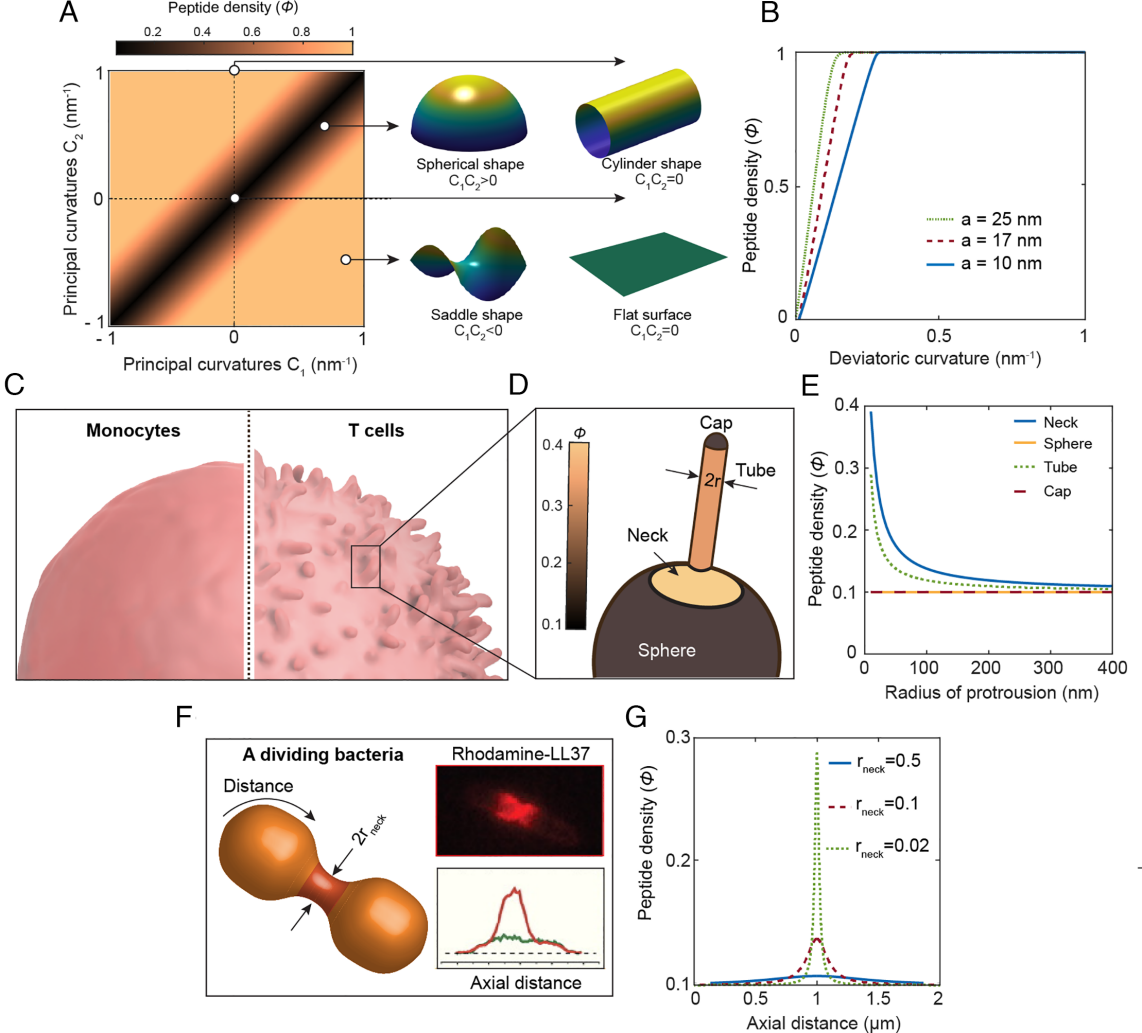
Mutations in SARS-CoV-2 Omicron variant reduce the pore-forming activity of xenoAMPs. (*A* and *B*) SAXS spectra of the SUV (DOPS/DOPE=20/80, P/L = 1/76) incubated with original xenoAMP(S) or Omicron xenoAMP(S). (*C*–*H*) flow cytometry measurement of xenoAMP(S)-induced cytotoxicity in human pDC, DCs, CD4^+^ T cells, CD8^+^ T cells, monocytes, and neutrophils. Peptide concentration used in *D*–*H* is 40 µM. Each point indicates an independent donor (The statistical analysis is done with one-way ANOVA, *P < 0.05, **P < 0.01).

The original xenoAMP(S) organizes the lipid membrane into a coexistence of Pn3m and Im3m cubic phases. The lattice constant in the Pn3m cubic phase is 17.0 nm (K = −0.023 nm^−2^) and the q ratios of the correlation peaks are √2: √3: √4: √8: √9: √10: √12: √14. The lattice constant in the Im3m cubic phase is 21.8nm (K = −0.023 nm^−2^). The q ratios of the correlation peaks are √2: √4: √6: √10. We compare this behavior to its Omicron homolog. Here, Omicron xenoAMP(S) is also capable of organizing lipid into Pn3m cubic phase (lattice constant is 19.2nm, K = −0.018 nm^−2^). The q ratio of the correlation peaks is √2: √3: √6: √9. The NGC observed in the cubic phase is smaller than that for the original xenoAMP(S) (Fig. 5*A* and *B*). AMPs with weaker capacity for inducing NGC tend to generate smaller and less stable transmembrane pores (20). Using a mean field model for estimating transmembrane pore sizes from cubic phase lattice constants based on membrane elasticity (20), we estimate that original xenoAMP(S) induces transmembrane pores with an average diameter between 1.46 to 1.86 nm, while the Omicron xenoAMP(S) induces generally smaller pores with an average estimated diameter from 1.32 to 1.74 nm (with 10% variation in membrane physical parameters). This general trend has been recapitulated in computer simulations. (*SI Appendix*, Fig. S3) Furthermore, the Omicron mutation responsible for the smaller induced pore is a single point mutation that increases the cationic charge of the peptide by +1. In general, increasing the cationic charge of an AMP sequence and/or reducing its hydrophobicity changes the AMP’s behavior toward that of a cell penetrating peptide (CPP), with typically smaller pores, shorter membrane residence times and shorter pore lifetimes (47) (a good extreme example is the CPP R9, with 9 arginines and no hydrophobic residues.). Taken together, these structural results suggest that the Omicron mutations, among other consequences, can potentially reduce the killing activity of SARS-CoV-2 derived xenoAMPs. We test this hypothesis on immune cells in the next section.

### XenoAMPs From Original SARS-CoV-2 Spike But Not Homolog xenoAMPs From the Omicron Spike Preferentially Kill the Immune Cells by Targeting the Negative Gaussian Membrane Curvature.

We investigate whether SARS-CoV-2 derived xenoAMPs can permeate the membrane of host immune cells and cause immune cell death, and whether the mutations introduced by the Omicron variants to the xenoAMPs affect their cytotoxicity. We used high concentrations of both the original xenoAMP(S) and its Omicron variant homolog (Omicron xenoAMP(S), T547K) as stringent test conditions to enable clear detection of potential immunotoxic effects. Cytotoxicity of both peptides are studied against six types of immune cells that are freshly isolated from the plasma of healthy human donor (Fig. 5*C*–*H*). PDCs and DCs are professional antigen-presenting cells and are early responders in viral infection. The depletion of pDCs is strongly associated with disease severity (2, 3). T cells, including CD4^+^ and CD8^+^ T cells are important effectors in the development of pathogen-specific adaptive immunity. It has been recently established that T cell lymphocytopenia is closely associated with COVID-19 disease severity (2, 48). Neutrophils and monocytes are professional phagocytes that can directly capture and degrade the viral particles. Neutrophils are highly activated in COVID-19 patients with acute respiratory distress syndrome (ARDS) (49, 50). Monocytes have been observed to undergo a slow maturation process that has been related to the cytokine dysregulation (51). These cells exhibit diverse membrane morphologies, which is ideal to study the curvature sensing preference of the xenoAMPs.

The isolated pDCs were incubated with increasing concentrations of either xenoAMP(S) or Omicron xenoAMP(S). Flow cytometry analysis revealed concentration-dependent cytotoxicity (Fig. 5*C*): XenoAMP(S) increased pDC death by 5.2-fold (0.3 µM), 25.7-fold (3 µM), and 68.3-fold (30 µM) compared with untreated healthy cell, while Omicron xenoAMP(S) induced much lower cell death (8.1-fold cell death increase at peptide concentration of 30 µM). Consistent with this finding, the study with DC, CD4^+^, and CD8^+^ T cells suggest xenoAMP(S) exhibits strong and acute cytotoxicity, increasing the number of dead cells by 3.2-fold, 6.4-fold, and 5.4-fold, respectively, compared to the PBS control. (Fig. 5 *D*–*H*) Interestingly, no significant cytotoxicity was observed in monocytes and neutrophils. In contrast, the Omicron xenoAMP(S) does not exhibit strong toxicity toward almost all of the immune cells studied, except for monocytes.

These results recapitulate the NGC sensing and xenoAMP accumulation behavior shown in Fig. 4. Immune cells with more drastic morphological projections on their cell surfaces and therefore display more NGC are more sensitive to xenoAMP-mediated killing. DCs and the subtype pDCs are both classical antigen-presenting cells. They have star-shaped morphologies due to their pseudopodia, which increases their surface area for antigen capture and presentation to lymphocytes. T cells (both CD4^+^ and CD8^+^ cells) surface is enriched with microvilli that become more pronounced after T cell activation. The base of pseudopodia and microvilli are rich in NGC and therefore attract NGC-generating sequences such as xenoAMPs, the local accumulation of which can promote pore formation and cell death. In contrast, monocytes and neutrophils that are less sensitive to xenoAMP toxicity exhibit smoother surfaces with less NGC. The mechanistic insights gained from these circulating model immune cells are directly applicable to tissue-resident immune populations, as they maintain conserved, cell-type-specific morphological and functional characteristics.

## Discussion

The mechanisms by which SARS-CoV-2 infection dysregulates key aspects of protective immunity have been largely unexplained. Depletion of specific immune cells, along with maladaptive cytokine profiles are hallmark of severe or long COVID-19 cases (1–3). The current study builds upon our recent discovery (5) that SARS-CoV-2 proteomes contain xenoAMP sequences distinct from their common cold coronavirus relatives, and which can induce dysregulated immune responses. Here, we show that direct proteolytic digestion of the SARS-CoV-2 spike proteins by trypsin-like serine proteases from the host immune system can produce viral fragments with the membrane remodeling activity cognate to AMPs, namely the ability to induce NGC and thereby form transmembrane pores. We demonstrate that this NGC inducing activity is common to xenoAMPs identified from different SARS-CoV-2 proteins. Importantly we find that this AMP-like capacity for inducing pores is not restricted to single xenoAMP fragment, but preserved even for the heterogeneous ensemble of xenoAMPs, non-xenoAMPs fragments, and endogenous AMP. This suggests that peptide fragments from viral proteomes with high concentrations of cationic amino acids, appropriately distributed, can in principle collectively increase the statistical propensity for pore formation.

Our computational modeling highlights the notion that while the peptides’ intrinsic physicochemical properties drive their NGC-sensing behavior, membrane physiochemical properties such as the lipid composition and net charge of the target membrane can also influence the ability of xenoAMPs to bind and remodel the membrane. For example, previous studies have shown that electrostatic interactions can play an important role in peptide–membrane association, especially for cationic peptides interacting with anionic lipid headgroups (52, 53). These factors are likely to modulate the propensity for pore formation by influencing peptide binding affinity, membrane insertion, and curvature stabilization.

A core tenet of our overarching hypothesis is that the heterogeneous immune dysregulation aligned with severity of COVID-19 is a consequence of the relevant enzyme proteome of the specific infected host. For example, gene dosing and polymorphisms of enzymes capable of degrading SARS-CoV-2 proteins are expected to render significant differences in peptide fragments even from the identical viral protein substrates. XenoAMPs generated by proteolytic cleavage can in principle be further cleaved in an infected host, with numbers that vary drastically among individuals due to heterogeneous enzymatic activity (22, 23). Therefore, it is interesting to compare results presented here to the clinical observation of depletion of pDCs, CD4^+^, and CD8^+^ T cells in severe COVID-19 patients, which are the exact cell types with complex morphologies that display high local levels of NGC. PDCs are potent secretors of type I (IFN-α and IFN-β) and type III interferons (IFN-λ), which are key cytokines for activating downstream interferon-stimulated genes that play important roles in antiviral responses. The delay of IFN-I secretion is positively correlated with delayed viral clearance and prolonging of inflammation, which polarizes monocytes and neutrophils into more proinflammatory phenotypes associated with off-target tissue injury (2, 54, 55). Likewise, the depletion of T cells also hinders the establishment of adaptive immunity, as T cells not only play regulatory roles in the maturation of B cells but can directly differentiate into cytotoxic effector T cells that recognize and clear virus infected cells (56). Based on differences in cell membrane composition and morphology, we expect the immune consequences of NGC targeting to be cell type specific and significant, since microvilli (and the associated NGC-rich regions) are more abundant in activated T cells that have already sensed viral infection. Indeed, depletion of T cell subsets is particularly detrimental to antiviral responses, as demonstrated by reduced CD8^+^ T cell threshold as a reliable biomarker of COVID-19 severity. Viral fragments from viruses that have been proteolytically destroyed can in principle continue to exert influence as xenoAMPs with consequences detrimental to host defense. For example, degradation of viral proteins leading to fragments that cripple host antiviral defenses may reflect the evolution of a viral strategy to subvert or circumvent innate and/or adaptive immune systems of complex hosts.

The results presented here offer a perspective on why the Omicron variant behaves differently compared to the native SARS-CoV-2 strain in certain human hosts, one distinct from viral replication and entry. Interestingly, the mutations introduced by the Omicron variant do not reduce the numbers of xenoAMPs harbored in SARS-CoV-2 proteins, but lead to different xenoAMP interactions with cell membranes. Our X-ray data from xenoAMPs derived from the Omicron spike suggest reduced levels of membrane-mediated killing activity relative to the original SARS-CoV-2 strain, consistent with our in vitro cell based experiments. Consistent with these observations, recent clinical observations show reduced CD8^+^ T cell cytopenia in individuals infected with Omicron variant (57). The conceptual framework here may also be relevant to other less virulent sublineages.

The potential emergence of a feedback loop between inflammation in COVID-19 patients and the production of xenoAMPs is worth noting. Our previous work indicates that the xenoAMPs derived from SARS-CoV-2 proteins can organize double-stranded RNA into liquid crystalline ordered complexes which amplify immune activation in multiple cell types, including monocytes, macrophages, epithelial cells, and endothelial cells, which exhibit a COVID-19 like transcriptome (5). Such inflammation can lead to the activation of trypsin-like serine protease activity (58), which increases the probability of creating more xenoAMPs (59). The increased production of xenoAMPs can in turn boost the inflammation and immune cell death in the host.

Recent work has identified the upregulation of host proteolytic activity as a significant immune consequence of SARS-CoV-2 infection. In vitro studies using bronchial epithelial cells show that infection upregulates multiple protease families, including endosomal cathepsins, secreted kallikrein-related peptidases (KLKs), and metalloproteases (60). Critically, this elevated protease expression coincides with a failure of antiprotease defenses in patients. Analyses of COVID-19 lung tissue reveal a suppressed antiprotease state, characterized by undetectable levels of α-1 antitrypsin (a major lung antiprotease) and downregulated antileukoprotease expression in both superficial epithelia and submucosal glands (58). This imbalance—increased protease expression alongside crippled antiprotease activity set the stage for strongly dysregulated proteolytic activity. The resulting proteolytic storm can in principle drive the increased production of viral protein fragments, including the membrane-active viral peptides described in our framework (5). This phenomenon aligns with the emerging understanding that proteolytic degradation of endogenous proteins constitutively generates AMP sequences as a component of the innate immune system.

Recent studies suggest remnant viral matter from SARS-CoV-2 and from other viruses persist in hosts for months after their infections have been cleared. SARS-CoV-2 spike proteins have been found to circulate in patients’ blood for months in the patients with myocarditis. Persistence of viral matter in the host has been hypothesized to play a role in chronic inflammation and long COVID. From the standpoint of the work here, these viral proteins can also serve as a persistent reservoir for xenoAMP generation. This may provide a possible explanation as to why the depletion of pDCs can persist for months after the initial COVID-19 infection (61). Together with our earlier work (5), the results here suggest that viral fragments may contribute significantly to the phenomenology of COVID-19. In a more general compass, we hypothesize that there exists a virtually unexplored layer of host–pathogen interactions mediated by proteolytic processing of proteins from viruses and microbes.

## Materials and Methods

Peptides or motifs with AMP-like physiochemical properties (xenoAMPs) were selected with a previously published AMP classifier (9). Mass spectrometry experiments were done in accordance with previous work by us (5), as were antimicrobial activity assays (5) and cytotoxicity assays (11). SAXS measurements were done at Stanford Synchrotron Radiation Lightsource (Beamline 4-2) and Shanghai Synchrotron Radiation facility (BL19U2). Theoretical analysis using continuum membrane elasticity model were performed in a manner cognate to our previous work (20). Molecular dynamics simulations were performed using GROMACS 2018.3. The human peripheral mononuclear cells (PBMCs) were collected from the venous blood drawn from healthy volunteers. Blood drawing was approved by the Ethikkommission der Ärztekammer Westfalen-Lippe of the University of Münster under the file number 2021-424-f-S. All donors were educated by medical staff before signing the informed consent. Human peripheral blood pDCs was collected by Blood Center of Policlinico Umberto I, Rome, IT, following approval by the ethics committee of Istituto Superiore di Sanità (ISS) of Rome (IT) (protocol number: 0008160). All samples are anonymized. Full details concerning materials and methods have been incorporated into *SI Appendix* in order to accommodate the multidisciplinary nature of this work.

## Supplementary Material

Appendix 01 (PDF)

## Data Availability

All data generated and/or analysed have been included in the article and/or *SI Appendix*.
