# Supplementary material for: SARS-CoV-2 peptide fragments selectively dysregulate specific immune cell populations via Gaussian curvature targeting
Source: Proc Natl Acad Sci U S A. 2026 Jan 8;123(2):e2521841122. doi: 10.1073/pnas.2521841122 (PMC12799121; doi:10.1073/pnas.2521841122)
Supplement: Supplementary file 1 — Appendix 01 (PDF) [file pnas.2521841122.sapp.pdf]

PNAS

Supporting Information for
SARS-CoV-2 peptide fragments selectively dysregulate specific immune cell populations via Gaussian curvature targeting

Yue Zhang[1,2,3,4,5,6,*], Carlos Silvestre-Roig[7,†], Han Fu[5,6,†], Haleh Alimohamadi[1,2,3,4,†], Taraknath Mandal[8,9,10], Jonathan W. Chen[1,2,3,4], Elizabeth Wei-Chia Luo[1,2,3,4], Jaime de Anda[1,2,3,4], Anna Lívia Linard Matos[7], Mathis Richter[7], HongKyu Lee[11], Liana C. Chan[11], Yingrui Wang[12,13,14], Naixin Wang[15], Hongyu Wang[5,6], Xiaohan Wang[6,14,16,17], Calvin K. Lee[1,2,3,4], Susmita Ghosh[18], Tsutomu Matsui[19], Thomas M. Weiss[19], Tiannan Guo[12,13,14], Maomao Zhang[15], Dapeng Li[6,14,16,17], Matthew C Wolfgang[20,21], Robert S. Hagan[21,22], Melody M.H. Li[3], Matthias Gunzer[18,23], Albert Sickmann[18,24], Loredana Frasca[25], Michael R. Yeaman[11,26,27,28], Roberto Lande[25], Qiang Cui[8,9], Oliver Soehnlein[7], Gerard C.L. Wong[1,2,3,4,*]

[1]Department of Bioengineering, University of California, Los Angeles; CA, 90095, USA; Los Angeles, CA, 90095, USA.

[2]Department of Chemistry and Biochemistry, University of California, Los Angeles; Los Angeles, CA, 90095, USA.

[3]Department of Microbiology, Immunology & Molecular Genetics, University of California, Los Angeles; Los Angeles, CA, 90095, USA.

[4]California NanoSystems Institute, University of California, Los Angeles; Los Angeles, CA, 90095, USA.

[5]School of Engineering, Westlake University; Hangzhou, Zhejiang, 310012, China.

[6]Research Center for Industries of the Future, Westlake University; Hangzhou, Zhejiang， 310024, China.

[7]Institute of Experimental Pathology (ExPat), Center for Molecular Biology of Inflammation (ZMBE), University Hospital Münster, University of Münster; Münster, 48143, Germany.

[8]Department of Chemistry, Boston University; Boston, MA 02215, USA.

[9]Department of Physics and Department of Biomedical Engineering, Boston University; Boston, MA 02215, USA.

[10]Department of Physics, Indian Institute of Technology Kanpur; Kanpur, 208016, India.

[11]Division of Molecular Medicine, Harbor-University of California Los Angeles Medical Center; Los Angeles County, Torrance, CA 90502, USA.

[12]Affiliated Hangzhou First People's Hospital, Westlake University; Hangzhou, Zhejiang, 02215, China.

[13]State Key Laboratory of Medical Proteomics; Hangzhou, Zhejiang, 02215, China.

[14]School of Medicine, Westlake University; Hangzhou, Zhejiang, 02215, China.

[15]Department of Cardiology, The Second Affiliated Hospital of Harbin Medical University, Harbin Heilongjiang, 150081, China.

[16]School of Life Sciences, Westlake University; Hangzhou, Zhejiang, 310012, China.

[17]Center for Infectious Disease research, Westlake University; Hangzhou, Zhejiang， 310024, China.

[18]Leibniz-Institut für Analytische Wissenschaften – ISAS – e.V.; Dortmund, 44139, Germany.

[19]Stanford Synchrotron Radiation Lightsource, SLAC National Accelerator Laboratory, Stanford University; Menlo Park, CA, 94025, USA.

[20]Department of Microbiology & Immunology, University of North Carolina School of Medicine; Chapel Hill, NC 27599, USA.

[21]Marsico Lung Institute, UNC School of Medicine, University of North Carolina at Chapel Hill; Chapel Hill, NC 27599, USA.

[22]Division of Pulmonary Diseases and Critical Care Medicine, UNC School of Medicine, University of North Carolina at Chapel Hill; Chapel Hill, NC 27599, USA;

[23]Institute for Experimental Immunology and Imaging, University Hospital, University of Duisburg-Essen; Essen, 45141, Germany.

[24]Medizinisches Proteom-Center, Ruhr-Universität Bochum; Bochum, 44801, Germany.

[25]National Center for Global Health, Istituto Superiore di Sanità (ISS); Rome, 00161, Italy.

[26]Division of Infectious Diseases, Harbor-University of California Los Angeles Medical Center; Los Angeles County, Torrance, CA 90502, USA.

[27]Department of Medicine, David Geffen School of Medicine, University of California, Los Angeles; CA 90095, USA.

[28]Institute for Infection & Immunity, Lundquist Institute for Biomedical Innovation, Harbor-University of California Los Angeles Medical Center; Torrance, CA 90502, USA.

† These authors contributed equally to this work.

***Corresponding authors:**

Yue Zhang, email: zhangyue59@westlake.edu.cn

Gerard C.L. Wong. email: gclwong@seas.ucla.edu

This PDF file includes:

Materials and Methods
Figure S1 to S3
Supplemental Table S1 to S3
SI References

Supporting Information

Supporting Materials and Methods

Recombinant spike protein production. Plasmids encoding SARS-CoV-2 variants with spike (S)-protein D614G mutations were produced by transiently transfecting human cell Expi293F™ Cells (ThermoFisher, Catalog no. A14527) using PEI MAX - Transfection Grade Linear Polyethylenim (Polysciences, Catalog no. 24765-1) at a final density of 2.5 x 10^7 viable cells/mL. The constructs contained a trimer tag followed by an HRV 3C-celavable C-terminal twinStrepTagII-8x His tag. On day 5, cell-free culture supernatant was generated by centrifugation of the culture and filtering through a 0.22-μm filter. Protein was purified from filtered cell culture supernatants by HisTrap HP, 5 x 5 ml (Cytiva) and by size-exclusion chromatography using HiLoad 16/600 Superose 6 pg size exclusive chromatography column (Cytiva) in 10 mM Tris pH8,500 mM NaCl.

Mass spectrometry analysis to peptide fragments. To investigate the peptides released from the SARS-CoV-2 spike protein following proteolytic digestion, recombinant full-length spike protein or the S1 subunit (Sino Biological) was initially denatured in 8 M urea (Sigma-Aldrich) within a 100 mM triethylammonium bicarbonate (TEAB, Sigma-Aldrich) buffer. The protein was then reduced with 10 mM tris (2-carboxyethyl) phosphine (TCEP; Adamas-beta) and alkylated with 40 mM iodoacetamide (IAA; Sigma-Aldrich). The recombinant spike protein or the S1 subunit was incubated with Lys-C (Hualishi Tech, #HLS LYS001C), neutrophil elastase (Abcam, #ab280938), or cathepsin G (Merck, #219373) at 32°C for 4 hours. Each digestion reaction contained 53 picomoles of spike protein or S1 subunit and 6 picomoles of protease in a final volume of 140 μL. The reaction was terminated by adding 10% trifluoroacetic acid (TFA; Thermo Fisher Scientific). Salt was removed from the samples using a Thermo Fisher C18 spin column (catalog number: 87782). To investigate if the peptides show AMP-like membrane affinity, the peptides were incubated with small unilamellar vesicles (SUV) or buffer control before loaded onto the C18 spin column. After desalting, the peptides were then eluted, dried, and redissolved for further analysis.

The peptide fragment analysis was performed with liquid chromatography-coupled tandem mass spectrometry (LC-MS/MS, Orbitrap 480, Thermo Fisher Scientific, San Jose, USA). The fragments from cathepsin G digested spike protein S1 subunit was analyzed with Exploris 480. The selection of peptide ion was controlled by a data dependent acquisition mode and the signal was analyzed by a label-free relative quantification approach. Mass spectrometric raw data was analyzed using Proteome Discoverer (Version 2.5.0.400, Thermo Fisher Scientific) by searching against the spike protein of SARS-CoV-2 virus FASTA downloaded from Uniprot (downloaded in June 2023). The cathepsin G result was analyzed with Proteome Discoverer 3.1.

The proteomic results of COVID-19 patient's sputum samples were adapted from a published study(1). The abundance of Kallikrein-1 (KLK1), KLK7, KLK10, KLK11, KLK12, KLK13, KLK14, KLKB1, elastase, cathepsin G and protease 3 were calculated by summing up the intensity of all peptide fragments detected from each protease.

XenoAMPs prediction. Peptides or motifs with AMP-like physiochemical properties (xenoAMPs) were selected with a previously published AMP classifier(2). This classifier uses a framework based on support vector machines (SVM). To predict the xenoAMPs motifs in SARS-CoV-2 (GenBank accession ID: MN938384), each protein is first scanned with a moving window to *in silico* generate sequences with length ranging from 24-34 amino acids. The classifier then assigned a σ score to each sequence to indicate its probability of being an AMP. A σ score > 0.4 suggests a probability > 79.4%, while a negative σ score suggests a low probability (P (+1) < 0.50). The AMPness of the identified peptides in the proteolytic digested spike protein is evaluated using the same method.

Peptide synthesis. All the peptides were custom synthesized by Lifetein. (Purity: 95%, C-terminal amidation and trifluoroacetic acid removed). The synthesized sequences include:

Original xenoAMP(S): KSTNLVKNKCVNFNFNGLTGTGVLTESNKK-NH_2 (Mw: 3255.25g/mol, locating at 529-558 in SARS-CoV-2 spike protein).

Omicron xenoAMP(S): KSTNLVKNKCVNFNFNGLKGTGVLTESNKK- NH_2 (Mw: 3283.76g/mol, the homolog sequence of xenoAMP(S) locating at 526-555 in SARS-COV-2 BA.5 spike protein).

Original xenoAMP(445-462): VGGNYNYLYRLFRKSNLK- NH_2 (Mw: 2204.18 g/mol)

Omicron xenoAMP(445-462): VGGNYNYRYRLFRKSNLK- NH_2 (Mw: 2247.20 g/mol)

xenoAMP(538-558): CVNFNFNGLTGTGVLTESNKK-NH_2 (Mw: 2242.10 g/mol)

xenoAMP(815-835): RSFIEDLLFNKVTLADAGFIK-NH_2(Mw: 2396.31 g/mol)

xenoAMP(948-964): LQDVVNQNAQALNTLVK-NH_2 (Mw: 2361.70g/mol).

Antimicrobial radial diffusion assay (RDA). The antimicrobial activity of SARS-CoV-2 xenoAMP(S) was assessed at both pH 7.5 and 5.5 using RDA. Three representative bacterial strains and one yeast strain were selected for this study: *E.faecalis* (29212), *A. baumannii* (HUMC 1), *K.pneumoniae* (NDM 1), *C. albicans* (36082R). A typical testing procedure is the following: Fresh colonies were picked from LB agar plates and transferred to liquid LB broth, then cultured overnight at 37 °C with shaking until reaching stationary phase. These cultures were diluted 1:100 into fresh LB broth and incubated at 37 °C for 2–3 hours until reaching mid-log phase (OD600 = 0.4-0.6). Subsequently, 10^6 CFU/mL of bacteria were evenly inoculated onto warm, liquid buffered molecular-grade agarose plates adjusted to pH 7.5 (PIPES buffer) or pH 5.5 (MES buffer). After solidification, 10 μg of xenoAMP(S) was introduced into wells of the seeded matrix and incubated at 37 °C for 3 hours, followed by the application of a nutrient agar overlay medium. The plates were then incubated at 37 °C for 24 hours before measuring the diameter of the zones of inhibition. The reported diameters represent the averages from four independent experiments.

Small unilamellar vesicles (SUVs). The SUV were prepared following the published protocol (3, 4). The SUVs mimicking lipid composition of eukaryotic cell membrane was prepared using sonication method. 1,2-dioleoyl-sn-glycero-3-phospho-L-serine (sodium salt, DOPS), 1,2-dioleoyl-sn-glycero-3-phosphoethanolamine (DOPE) and cholesterol were all purchased from Avanti. Two lipid compositions were made: A ternary mixture of DOPS, DOPE and cholesterol in a molar ratio of 20:70:10. And a binary mixture of DOPS and DOPE at a molar ratio of 20:80. The lipid powder was first dissolved in chloroform and mixed at the designed ratio before dried under the nitrogen gas for 10mins. The residual chloroform was completely removed by placing the lipid sample in desiccator overnight. PH7.4 10mM HEPES buffer containing 140mM NaCl was added to the lipid composition to fully rehydrate the lipid. SUV was formed by sonicating the rehydrated lipid for 10mins. The SUV was then extruded through 0.22μm filter to remove the debris. Lipid composition was stored at 4 ℃ for further experiment.

Small angle X ray scattering (SAXS). The membrane activity of peptide fragments released from proteolytically degraded spike protein was assessed using SAXS. For trypsin digestion, 70 picomoles of spike protein were incubated with 0.4 picomoles of trypsin (Thermo Scientific, 20233) in a final volume of 120 μL. For KLK5 digestion, the spike protein was incubated with KLK5 (BioLegend, #790204) at a 3:1 protein: protease molar ratio. The reaction was carried out at 37°C for 4 hours, followed by heat inactivation at 65 °C for 10mins. The mixture was then incubated with SUVs at the designed protein-to-lipid molar ratio. To study the membrane remodeling activity of the synthetic xenoAMPs, the peptides were directly mixed with SUV at the designated molar ratio. The sample was left on the shaker overnight before loaded into the quartz capillaries (diameter = 1.5mm, Hilgenberg GmbH). All samples were kept at 37°C overnight before SAXS measurement. SAXS experiments evaluating peptides' membrane activity were performed at Stanford Synchrotron Radiation Lightsource (SSRL, Beamline 4-2) using monochromatic X-rays with the wavelength λ =1.378 Å (energy 9keV). A Pilatus3 X 1M detector was used to collect the scattering signal. SAXS experiments were repeated at Shanghai Synchrotron Radiation Facility (SSRF, Beamline BL19U2) using monochromatic X-rays with the wavelength λ =1.033 Å (energy 12keV). The scattering signal was collected with Pilatus 2M detector. The two-dimensional powder diffraction pattern was integrated with Nika package 1.81 in Igor Pro 7. The q ratio of Pn3m is $\sqrt{2}:\sqrt{3}:\sqrt{4}:\sqrt{6}:\sqrt{8}:\sqrt{9}:\sqrt{10}:\sqrt{11}:\sqrt{12}:\sqrt{14}:\sqrt{16}:\sqrt{17}$. The q ratio of Im3m is $\sqrt{2}:\sqrt{4}:\sqrt{6}:\sqrt{8}:\sqrt{10}:\sqrt{12}:\sqrt{14}:\sqrt{16}$. The average amount of Gaussian curvature(K) in a cubic phase is be calculated using this equation $\langle K \rangle = (2\pi\chi)/(A_0 a^2)$, where a is the lattice constant of cubic phase. The Euler characteristic, χ, and surface area per unit cell, A_0, are constants unique to each cubic phase. For Pn3m, $\chi = -2$ and A0 = 1.919, and for Im3m, $\chi = -4$ and $A_0 = 2.345$.

Computer simulation of pore formation. Atomistic model of original xenoAMP(S) (PDB ID: 6VSB), the Omicron xenoAMP(S) (PDB ID: 7TGW) and the N terminus of histone H4 (PDB ID: 1KX5) were downloaded from the protein data bank. Nine copies of the peptides were placed randomly on top of a lipid bilayer consisting of 160 DOPC and 40 DOPS lipids, atomistic models of which were generated using CHARMM-GUI(5, 6). The peptide-membrane composite system was then solvated using TIP3P water(7). Appropriate number of Na^+ and Cl^- ions were added to achieve charge neutrality. CHARMM36(8-10) force field was used to model the lipids, peptides, water and ions. The hydrogen atoms were constrained using the LINCS algorithm(11) which allowed a time step of 2 fs for integrations. The built structure was energy minimized, first using the steepest decent method, and followed by the conjugate gradient method to remove any bad contacts between the solute and solvent atoms. This was followed by a 30-ns long simulation in which the lipid bilayer was described using highly mobile membrane mimetic model (HMMM)(12) to achieve rapid equilibration of local lipid distributions around the peptides. The equilibrated HMMM structures were then converted to full lipid model using CHARMM-GUI(13) (5, 6). The peptide-membrane composite system was then solvated using TIP3P water(7). Appropriate number of Na^+ and Cl^- ions were added to achieve charge neutrality. CHARMM36(8-10) force field was used to model the lipids, peptides, water and ions. The hydrogen atoms were constrained using the LINCS algorithm(11) which allowed a time step of 2 fs for integrations. The built structure was energy minimized, first using the steepest decent method, and followed by the conjugate gradient method to remove any bad contacts between the solute and solvent atoms. This was followed by a 30-ns

long simulation in which the lipid bilayer was described using highly mobile membrane mimetic model (HMMM)(12) to achieve rapid equilibration of local lipid distributions around the peptides. The equilibrated HMMM structures were then converted to full lipid model using CHARMM-GUI(13). These full lipid and peptide complex systems were then simulated using constant pressure, constant area (NPAT) ensemble in which the area per lipid was taken approximately 50% higher than its equilibrium value which facilitates water channel formation by the peptides across the membrane. Two different initial configurations were generated from the NPAT simulation trajectory. Each of the systems was then simulated in restrain-free constant-pressure (1 bar) constant-temperature (303K) simulations for 500 ns during which the box lengths spontaneously readjusted to achieve the correct lipid density and a stable water channel in the membrane within approximately the first 25 ns. The temperature and pressure of the system was controlled by a Nose-Hoover thermostat(14, 15) with a time constant of 1 ps, and a Parrinello-Rahman barostat(16) with a time constant of 5 ps, respectively. The water channel formed by the peptides was found to be stable throughout the entire period in the 500 ns-long independent simulations; the stability of the water channel was assessed by monitoring the number of water molecules in the central part of the channel. Finally, a controlled simulation was performed in which the peptides were removed from a stable water channel and the peptide-free system was equilibrated. The water channel gradually started to shrink and ultimately disappeared within only 7 ns. The simulations were performed using the GROMACS-2018.3 software(17).(14, 15) with a time constant of 1 ps, and a Parrinello-Rahman barostat(16) with a time constant of 5 ps, respectively. The water channel formed by the peptides was found to be stable throughout the entire period in the 500 ns-long independent simulations; the stability of the water channel was assessed by monitoring the number of water molecules in the central part of the channel. Finally, a controlled simulation was performed in which the peptides were removed from a stable water channel and the peptide-free system was equilibrated. The water channel gradually started to shrink and ultimately disappeared within only 7 ns. The simulations were performed using the GROMACS-2018.3 software(17).

Membrane mechanics. We consider a system that includes a lipid membrane and embedded xenoAMPs on the membrane plane such that they can diffuse freely on the surface of the membrane. Using a continuum description, the total free energy density of the system (W) can be written as

$$W = W_m + W_\phi + W_I, \quad \text{(S1)}$$

where W_m is the elastic energy due to the mismatch between the local shape of the lipid membrane and induced curvature by peptides , W_ϕ is the entropy of the peptide diffusion in the plane of the membrane, and W_I is the peptide-peptide interaction energy. Assuming the lipid bilayer is a thin elastic shell with a negligible thickness, we modeled the membrane-peptide interactions energy using the modified version of Helfrich–Canham energy given by(18-26)

$$W_m = \kappa H^2 + \kappa(D - d_0)^2, \quad \text{(S2)}$$

where κ is the membrane bending modulus, $H = (C_1 + C_2)/2$ is the mean curvature ($C_1$and C_2 are the principal curvatures of the membrane at any given point), $D = (C_1 - C_2)/2$ is the curvature deviator, d_0 is the intrinsic (spontaneous) curvature deviator, and ϕ is the fraction of the membrane area covered by xenoAMPs. Here, we assume that the induced curvature deviator (d_0) depend linearly on the peptide area fraction (ϕ) as (27-30).

$$d_0 = D_0\phi, \quad \text{(S3)}$$

where D_0 is a constant representing the magnitude of induced curvatures by xenoAMPs. Using the principle of entropy maximization, the entropic component of the free energy can be written as(31)

$$W_\phi = k_B T n_s(\phi \log(\phi) + (1 - \phi)\log(1 - \phi)), \quad \text{(S4)}$$

where k_B is the Boltzmann constant, T is the absolute temperature, and n_s is the saturation density of peptides on surface. Using the Bragg-Williams approximation within mean field theory, the interaction energy between peptides on can be expressed as(29, 30, 32).

$$W_I = \frac{\gamma n_s}{2}\phi^2, \tag{S5}$$

where γ is the net effective interaction energy of the peptides, with $\gamma > 0$ representing the peptide-peptide attraction and $\gamma < 0$ indicating the peptide-peptide repulsion.

Substituting Eqs S2-S5 in Eq. S1 gives the total energy density as

$$W = \kappa H^2 + \kappa(D - D_0\phi)^2 + k_B T n_s(\phi\log(\phi) + (1-\phi)\log(1-\phi)) + \frac{\gamma n_s}{2}\phi^2. \tag{S6}$$

Assuming the system is in mechanochemical equilibrium, we can obtain the xenoAMPs distribution by minimizing the free energy density given as

$$m = \frac{\partial W}{\partial \phi} = \text{ const}, \tag{S7}$$

Here, m represents the peptide chemical potential on the surface. Substituting Eq. S6 into Eq. S7 and rearranging the terms, we have

$$\log\left(\frac{\phi}{1-\phi}\right) = -\left(\frac{2\kappa}{k_B T n_s}D_0^2 + \frac{\gamma}{\kappa_\beta T}\right)\phi + \frac{2\kappa}{k_B T n_s}DD_0 + const/k_B T n_s. \tag{S8}$$

The induced deviatoric curvature D_0 in Eq. S8 can be estimated based on the cubic structures observed in high-resolution synchrotron SAXS as (26, 33, 34)

$$D_0 = \langle D_{cubic}\rangle = \sqrt{\frac{2\pi\chi}{A^* a^2}}, \tag{S9}$$

where $\langle D_{cubic}\rangle$ is the average membrane curvature deviator in a cubic phase, a is the cubic lattice parameter, χ is the Euler characteristic, and A^* is the surface area per unit cell which are constants specific to each cubic phase. Here, at the low peptide concentrations, we assume the effective peptide-peptide interaction energy is weak ($\gamma < \kappa_\beta T$)(35). Previous studies have also suggested that AMPs such as magainin and melittin interact indirectly via membrane-mediated elasticity (e.g., membrane thinning and induced curvature) rather than through strong direct interactions(36, 37). Thus, within the weak interaction regime $|\gamma| \ll |2\kappa D_0^2/n_s|$, the curvature mismatch term in the free energy density dominates and Eq. 8 simplifies as

$$\log\left(\frac{\phi}{1-\phi}\right) = -\frac{2\kappa}{k_B T n_s}D_0^2\,\phi + \frac{2\kappa}{k_B T n_s}DD_0 + const/k_B T n_s\,. \tag{S10}$$

For any given set of constant system parameters, we numerically solved Eq. S10 using MATLAB to find the xenoAMPs distribution based on the membrane geometry. For simplicity, we set $const$ =0. To fix the number of xenoAMPs on the membrane for spiky-shaped cells such as pDC and $CD4^+$ Tcells, we can use a Lagrange multiplier (λ) and rewrite Eq. S6 with no interaction energy as

$$W = \kappa H^2 + \kappa(D - D_0\phi)^2 + k_B T n_s(\phi\log(\phi) + (1-\phi)\log(1-\phi)) + \lambda\phi. \tag{S11}$$

Using the conservation equation for xenoAMPs on the membrane surface, we have

$$\int \phi dA = \phi_{average} A_0, \quad (S12)$$

where $\phi_{average}$ is the average value of ϕ and A_0 is the total area of the membrane. Eqs. S11 and S12 allow us to find the xenoAMPs distribution for a given membrane geometry. Biologically relevant values for the parameters that have been used in the mathematical model are listed in **Table S3**.

Cytotoxicity study. To isolate human peripheral blood plasmacytoid dendritic cells (pDCs), blood buffy coats of healthy donors (HD) were obtained from Blood Center of Policlinico Umberto I, Rome, IT, following approval by the ethics committee of Istituto Superiore di Sanità (ISS) of Rome (IT) (protocol number: 0008160). All samples are anonymized. After separation of PBMCs by Ficoll centrifugation, pDCs were purified by using Diamond Plasmacytoid Dendritic Cell Isolation Kit (Miltenyi Biotec, Gladbach, Germany). Cell purity was evaluated by staining the cells with anti-CD123-APC and anti-BDCA2-PE antibodies (Miltenyi Biotec, Gladbach, Germany) and flow cytometry acquisition. Purified pDCs were seeded into 96-well round-bottom plates at 400×10^3 cells ml^{-1}. Original xenoAMP(S) or Omicron xenoAMP(S) were incubated with pDC for 4h, together with recombinant human IL-3 (10 ng ml^{-1}; PeproTech, London, UK). At the end of the treatment, cells were washed with protein-free phosphate buffer saline (PBS, Lonza BioWhittaker) and stained with 100 µl of Fixable Viability Dye (FVD) eFluor780 (Invitrogen, Thermo Fisher Scientific) diluted 1:1000 in PBS, 30 minutes at 4 °C. Cells were then acquired by flow cytometry (Gallios, Beckman Coulter) and analyzed by FlowJo 10.0.7 (Tristar, USA).

To isolate human peripheral mononuclear cells (PBMCs), venous blood was drawn from healthy volunteers. Blood drawing was approved by the Ethikkommission der Ärztekammer Westfalen-Lippe of the University of Münster under the file number 2021-424-f-S. All donors were educated by medical staff before signing the informed consent. All samples are anonymized. PBMCs and granulocytes were isolated using density centrifugation PolymorphPrep (Progen). Dendritic cells, T-cells and Monocytes were further purified using Pan-dendritic cell, Pan-T cell, and Pan-monocyte isolation kits from Miltenyi, respectively. Red blood cells were lysed from isolated cell subpopulations using RBC lysis buffer (Biolegend). Isolated blood leukocytes were incubated with indicated antibodies (see below) in cell staining buffer (Biolegend) for 20 min on ice. After washing, stained cells were acquired using a 5 lasers Cytek Aurora system. Dendritic and T-cells were incubated with an antibody mix containing CD11b-PerCPCy5.5, CD62L-PE, CD15-BUV805, CD16-BUV496, HLA-DR-BUV661, CD14-BV605, CD4-Pacific blue, CD3-Spark Blue 550, CD8-APC-H7, CD45RA-BV570, CD11C-BV785, CD304-BV510, CD123-APC-Fire-810, CD19-PE-Dazzle 594 and CD79b-BV711. Neutrophils and monocytes were incubated with an antibody mix containing CD11b-PerCPCy5.5, CD62L-PE, CD15-BUV805, CD16-BUV496, HLA-DR-BUV661, CD14-BV605, CD10-BUV395, CD182-BV650, CD66b-FITC, CD101-PE-Vio770. All fluorescent antibodies were purchased from Biolegend, BD Biosciences or Miltenyi. The isolated blood leukocytes were incubated with original xenoAMP(S), Omicron xenoAMP(S) or PBS at 40µM for 4 hours at 37°C in RPMI (no serum). Later, cells were washed with HBBS 1x + 0.05mM EDTA + 0.5% BSA at 4°C. Cells were then incubated with fluorescent antibodies and non-permeable DAPI (Invitrogen, 5 ng/mL). The viability was then analyzed with flow cytometry.

Figures

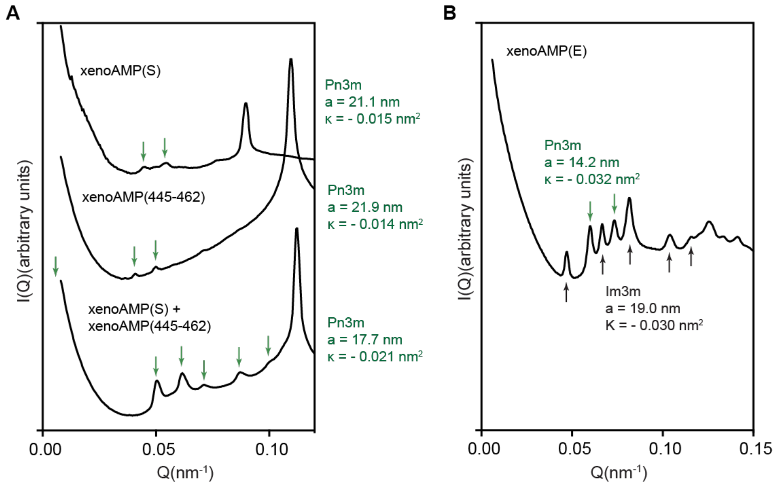

Figure S1. SAXS analysis of xenoAMP-induced membrane remodeling activity. (A) Small unilamellar vesicles (SUVs) composed of DOPS/DOPE/cholesterol (20:70:10 as molar ratio) were incubated with either individual xenoAMPs or a mixture of xenoAMP(S) and xenoAMP(445-462) at a peptide-to-lipid ratio (P/L) of 1:38. While all samples formed Pn3m cubic phases, the xenoAMP mixture induced a *single* cubic phase with greater negative Gaussian curvature (NGC, κ) compared to those from individual xenoAMPs. This shows that heterogeneous mixtures of xenoAMPs can cooperatively form pores. (**B**) xenoAMP derived from SARS-CoV-2 envelope protein can remodel membrane into two coexisting cubic phases: Pn3m and Im3m. (P/L = 1/77)

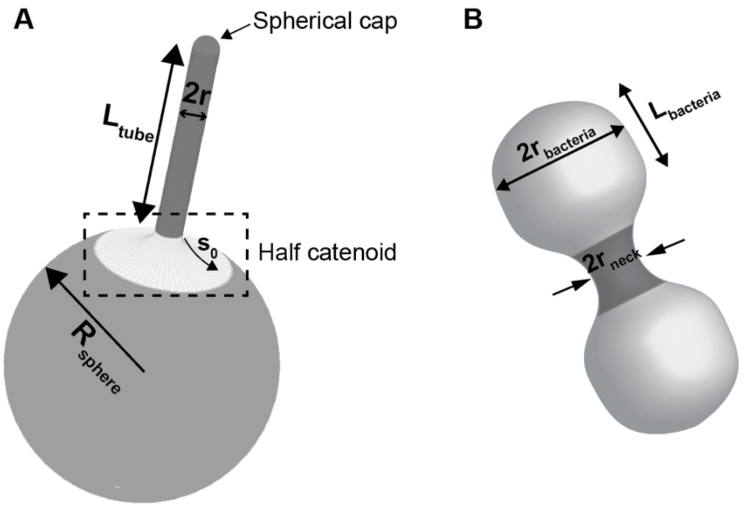

Figure S2. (**A**) Idealized geometry of a dendritic cell including a spherical body with radius R_{sphere} connected via a half catenoid-shaped neck to a tubular protrusion with radius r and length L_{tube}. The tubular protrusion is closed with a spherical cap and the length of the half catenoid is s_0. **(B)** Idealized geometry of a dividing bacterial cell including two cylinders with a radius of $r_{bacteria}$ and length of $L_{bacteria}$ connected to each other via a catenoid-shaped neck.

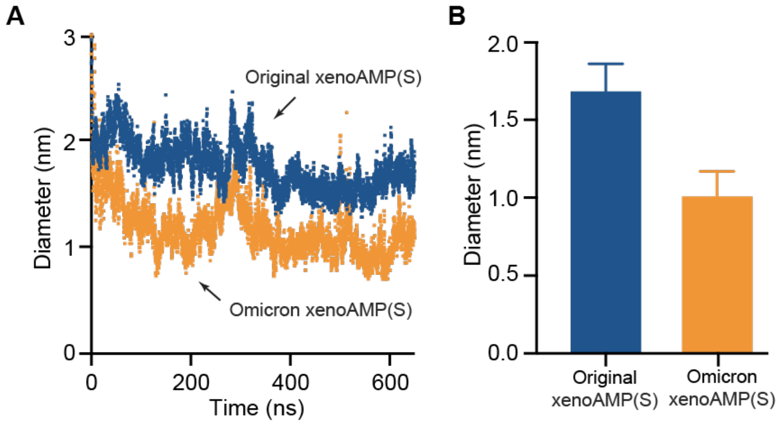

Figure S3. MD simulation of xenoAMPs-induced pore formation. Omicron xenoAMP(S) carry a single mutation (T547K) that increases the cationic charge of the peptide. The diameter of the transmembrane pore formed by Omicron xenoAMP(S) is smaller than that of the original xenoAMP(S). Average diameters are calculated from two independent simulations in both cases.

Tables

Table S1. Peptides identified from proteolytically digested full length spike protein or S1 subunit.

LysC-digested spike protein

xenoAMP sequences	Position	σ score	Intensity
[K].NLREFVFKNIDGYFK.[I]	188-202	0.33	193.9
[K].VGGNYNYLYRLFRK.[S]	445-458	0.47	104.6
[K].CVNFNFNGLTGTGVLTESNK.[K]	538-557	0.42	165.1
[K].CVNFNFNGLTGTGVLTESNKK.[F]	538-558	0.6	500
[K].RSFIEDLLFNK.[V]	815-825	2.66	125.9
[K].RSFIEDLLFNKVTLADAGFIK.[Q]	815-835	1.19	343.9
[K].QYGDCLGDIAARDLICAQK.[F]	836-854	0.2	96.3
[K].LIANQFNSAIGK.[I]	922-933	0.19	84
[K].LIANQFNSAIGKIQDSLSSTASALGK.[L]	922-947	0.42	157.9
[K].IQDSLSSTASALGK.[L]	934-947	0.45	79.7
[K].LQDVVNQNAQALNTLVK.[Q]	948-964	0.34	77.3
[K].QLSSNFGAISSVLNDILSRLDK.[V]	965-986	0.78	452.1

Elastase-digested spike protein

xenoAMP sequences	Position	σ score	Intensity
[L].EGKQGNFKNLREFV.[F]	180-193	0.21	100.7
[V].GGNYNYLYRLFRKSNL.[K]	446-461	0.18	132.6
[V].LTESNKKFLPFQQFGRDI.[A]	552-569	1.04	69.8
[V].LTESNKKFLPFQQFGRDIA.[D]	552-570	0.29	76.2
[L].TESNKKFLPFQQFGRDIA.[D]	553-570	0.41	92.2
[I].EDLLFNKV.[T]	819-826	2.23	64.5
[A].NQFNSAIGKI.[Q]	925-934	0.02	101.7

Cathepsin G-digested S1 subunit

xenoAMP sequences	Position	σ score	Intensity
[F].ASTEKSNIIRGWIF.[G]	93-106	0.05	2.99×10^7
[F].KIYSKHTPINLVRDLPQGF.[S]	202-220	0.56	8.08×10^6
[Y].SKHTPINLVRDLPQGF.[S]	205-220	0.06	2.89×10^6
[F].NGLTGTGVLTESNKKFLPF.[Q]	544-562	0.77	1.35×10^7
[L].TESNKKFLPFQQF.[G]	553-565	0.44	1.10×10^6

Table S2. Antimicrobial activity of SASR-CoV-2 xenoAMP(S)

Diameter of zone of clearning (mm, n = 4)			Species
Microbes	pH7.5	pH5.5	
E. faecalis	1.69 ± 0.14	4.33 ± 0.24	Gram positive bacteria
A. baumannii	6.37 ± 0.42	6.52 ± 0.82	Gram negative bacteria
K. pneumoniae	8.08 ± 0.88	5.22 ± 0.92	Gram negative bacteria
C. albicans	0	4.61 ± 2.28	Yeast

Table S3. Parameters used in the mechanical model

Parameter	Significance	Value
κ	Membrane bending rigidity	$10kT$ (38)
n_s	Saturation density of xenoAMPs on surface	0.04 nm^{-2}
$\phi_{average}$	Average peptide area fraction	0.1
s_0	Length of half catenoid	10 nm
χ	Euler characteristic for a *Pn3m*	-2 (33, 39)
A^*	Surface area per unit cell for a *Pn3m*	1.919 (33, 39)
L_{tube}	Length of the tubular protrusion	20 µm (40)
R_{sphere}	Radius of spherical body	20 µm (40)
$L_{bacteria}$	Length of the half of divided bacteria	2 µm(41)
$r_{bacteria}$	Radius of divided bacteria	1 µm (41)

SI References

1. J. Fisher *et al.*, Proteome Profiling of Recombinant DNase Therapy in Reducing NETs and Aiding Recovery in COVID-19 Patients. *Mol Cell Proteomics* **20**, 100113 (2021).
2. E. Y. Lee, B. M. Fulan, G. C. Wong, A. L. Ferguson, Mapping membrane activity in undiscovered peptide sequence space using machine learning. *Proc Natl Acad Sci U S A* **113**, 13588-13593 (2016).
3. C. Silvestre-Roig *et al.*, Externalized histone H4 orchestrates chronic inflammation by inducing lytic cell death. *Nature* **569**, 236-240 (2019).
4. M. W. Lee *et al.*, Apolipoprotein Mimetic Peptide Inhibits Neutrophil-Driven Inflammatory Damage via Membrane Remodeling and Suppression of Cell Lysis. *Acs Nano* **15**, 15930-15939 (2021).
5. S. Jo, T. Kim, V. G. Iyer, W. Im, CHARMM-GUI: a web-based graphical user interface for CHARMM. *J Comput Chem* **29**, 1859-1865 (2008).

6. S. Jo, J. B. Lim, J. B. Klauda, W. Im, CHARMM-GUI Membrane Builder for mixed bilayers and its application to yeast membranes. *Biophys J* **97**, 50-58 (2009).
7. W. L. Jorgensen, J. Chandrasekhar, J. D. Madura, R. W. Impey, M. L. Klein, Comparison of Simple Potential Functions for Simulating Liquid Water. *J Chem Phys* **79**, 926-935 (1983).
8. J. B. Klauda *et al.*, Update of the CHARMM All-Atom Additive Force Field for Lipids: Validation on Six Lipid Types. *J Phys Chem B* **114**, 7830-7843 (2010).
9. R. B. Best *et al.*, Optimization of the Additive CHARMM All-Atom Protein Force Field Targeting Improved Sampling of the Backbone φ, ψ and Side-Chain χ and χ Dihedral Angles. *J Chem Theory Comput* **8**, 3257-3273 (2012).
10. J. Huang, A. D. MacKerell, CHARMM36 all-atom additive protein force field: Validation based on comparison to NMR data. *Journal of Computational Chemistry* **34**, 2135-2145 (2013).
11. B. Hess, H. Bekker, H. J. C. Berendsen, J. G. E. M. Fraaije, LINCS: A linear constraint solver for molecular simulations. *Journal of Computational Chemistry* **18**, 1463-1472 (1997).
12. Y. Z. Ohkubo, T. V. Pogorelov, M. J. Arcario, G. A. Christensen, E. Tajkhorshid, Accelerating Membrane Insertion of Peripheral Proteins with a Novel Membrane Mimetic Model. *Biophysical Journal* **102**, 2130-2139 (2012).
13. Y. F. Qi *et al.*, CHARMM-GUI HMMM Builder for Membrane Simulations with the Highly Mobile Membrane-Mimetic Model. *Biophysical Journal* **109**, 2012-2022 (2015).
14. S. Nose, A Unified Formulation of the Constant Temperature Molecular-Dynamics Methods. *J Chem Phys* **81**, 511-519 (1984).
15. W. G. Hoover, Canonical Dynamics - Equilibrium Phase-Space Distributions. *Phys Rev A* **31**, 1695-1697 (1985).
16. M. Parrinello, A. Rahman, Polymorphic Transitions in Single-Crystals - a New Molecular-Dynamics Method. *J Appl Phys* **52**, 7182-7190 (1981).
17. T. M. Mark James Abraham, Roland Schulz , Szilárd Páll, Jeremy C. Smith, Berk Hess, Erik Lindahl, GROMACS: High performance molecular simulations through multi-level parallelism from laptops to supercomputers. *SoftwareX* **1-2**, 19-25 (2015).
18. A. Iglic, T. Slivnik, V. Kralj-Iglic, Elastic properties of biological membranes influenced by attached proteins. *J Biomech* **40**, 2492-2500 (2007).
19. N. Bobrovska, W. Gozdz, V. Kralj-Iglic, A. Iglic, On the role of anisotropy of membrane components in formation and stabilization of tubular structures in multicomponent membranes. *PLoS One* **8**, e73941 (2013).
20. V. Kralj-Iglic, V. Heinrich, S. Svetina, B. Zeks, Free energy of closed membrane with anisotropic inclusions. *Eur Phys J B* **10**, 5-8 (1999).
21. H. J. Deuling, W. Helfrich, Red Blood-Cell Shapes as Explained on Basis of Curvature Elasticity. *Biophysical Journal* **16**, 861-868 (1976).
22. W. Helfrich, Elastic Properties of Lipid Bilayers - Theory and Possible Experiments. *Zeitschrift Fur Naturforschung C-a Journal of Biosciences* **C 28**, 693-703 (1973).
23. H. Alimohamadi, P. Rangamani, Modeling Membrane Curvature Generation due to Membrane-Protein Interactions. *Biomolecules* **8** (2018).
24. N. Walani, J. Torres, A. Agrawal, Anisotropic spontaneous curvatures in lipid membranes. *Phys Rev E* **89** (2014).
25. P. B. Canham, The minimum energy of bending as a possible explanation of the biconcave shape of the human red blood cell. *J Theor Biol* **26**, 61-81 (1970).
26. H. Alimohamadi *et al.*, How Cell-Penetrating Peptides Behave Differently from Pore-Forming Peptides: Structure and Stability of Induced Transmembrane Pores. *J Am Chem Soc* **145**, 26095-26105 (2023).
27. J. Derganc, Curvature-driven lateral segregation of membrane constituents in Golgi cisternae. *Phys Biol* **4**, 317-324 (2007).
28. V. S. Markin, Lateral Organization of Membranes and Cell Shapes. *Biophysical Journal* **36**, 1-19 (1981).
29. H. Alimohamadi, B. Ovryn, P. Rangamani, Modeling membrane nanotube morphology: the role of heterogeneity in composition and material properties. *Sci Rep-Uk* **10** (2020).

30. N. S. Gov, Guided by curvature: shaping cells by coupling curved membrane proteins and cytoskeletal forces. *Philos Trans R Soc Lond B Biol Sci* **373** (2018).
31. S. Safran, *Statistical Thermodynamics Of Surfaces, Interfaces, And Membranes* (CRC Press, 2003), https://doi.org/10.1201/9780429497131.
32. S. Leibler, D. Andelman, Ordered and Curved Meso-Structures in Membranes and Amphiphilic Films. *J Phys-Paris* **48**, 2013-2018 (1987).
33. U. S. Schwarz, G. Gompper, Systematic approach to bicontinuous cubic phases in ternary amphiphilic systems. *Phys Rev E* **59**, 5528-5541 (1999).
34. P. E. Harper, S. M. Gruner, R. N. A. H. Lewis, R. N. McElhaney, Electron density modeling and reconstruction of infinite periodic minimal surfaces (IPMS) based phases in lipid-water systems. II. Reconstruction of D surface based phases. *Eur Phys J E* **2**, 229-245 (2000).
35. B. Honig, A. Nicholls, Classical Electrostatics in Biology and Chemistry. *Science* **268**, 1144-1149 (1995).
36. M. T. Lee, W. C. Hung, F. Y. Chen, H. W. Huang, Many-body effect of antimicrobial peptides: On the correlation between lipid's spontaneous curvature and pore formation. *Biophysical Journal* **89**, 4006-4016 (2005).
37. H. W. Huang, Elasticity of Lipid Bilayer Interacting with Amphiphilic Helical Peptides. *J Phys Ii* **5**, 1427-1431 (1995).
38. W. Rawicz, Olbrich, K. C., McIntosh, T., Needham, D., Evans, E. , Effect of chain length and unsaturation on elasticity of lipid bilayers. *Biophysical Journal* **79**, 328–339 (2000).
39. P. E. Harper, S. M. Gruner, Electron density modeling and reconstruction of infinite periodic minimal surfaces (IPMS) based phases in lipid-water systems. I. Modeling IPMS-based phases. *Eur Phys J E* **2**, 217-228 (2000).
40. G. Grouard *et al.*, The enigmatic plasmacytoid T cells develop into dendritic cells with interleukin (IL)-3 and CD40-ligand. *J Exp Med* **185**, 1101-1111 (1997).
41. K. A. Sochacki, K. J. Barns, R. Bucki, J. C. Weisshaar, Real-time attack on single Escherichia coli cells by the human antimicrobial peptide LL-37. *Proc Natl Acad Sci U S A* **108**, E77-81 (2011).
